# Illicit financial flows and the provision of child and maternal health services in low- and middle-income countries

**DOI:** 10.1186/s12914-020-00236-w

**Published:** 2020-07-11

**Authors:** Bienvenido Ortega, Jesús Sanjuán, Antonio Casquero

**Affiliations:** Departamento de Economía Aplicada (Estructura Económica), Universidad de Málaga, Campus El Ejido, 29071 Málaga, Spain

**Keywords:** Illicit financial flows, Health financing, Essential health services coverage, Family planning, Antenatal care, Child immunisation, Sustainable development goals, Low- and middle-income countries

## Abstract

**Background:**

Illicit financial flows (IFFs) drain domestic resources with harmful social effects, especially in countries which are too poor to mobilise the revenues required to finance the provision of essential public goods and services. In this context, this article empirically examined the association between IFFs and the provision of essential health services in low- and middle-income countries.

**Methods:**

Firstly, a set of indicators was selected to represent the overall coverage of essential health services at the country level. Next, a linear multivariate regression model was specified and estimated for each indicator using cross-sectional data for 72 countries for the period 2008–2013.

**Results:**

After controlling for other relevant factors, the main result of the regression analysis was that an annual 1 percentage point (p.p.) increase in the ratio of IFFs to total trade was associated with a 0.46 p.p. decrease in the level of family planning coverage, a 0.31 p.p. decrease in the percentage of women receiving antenatal care, and a 0.32 p.p. decrease in the level of child vaccination coverage rates.

**Conclusions:**

These findings suggest that, for the whole sample of countries considered, at least 3.9 million women and 190,000 children may not receive these basic health care interventions in the future as a consequence of a 1 p.p. increase in the ratio of IFFs to total trade. Moreover, given that family planning, reproductive health, and child immunisation are foundational components of health and long-term development in poor countries, the findings show that IFFs could be undermining the achievement of the 2030 Agenda for Sustainable Development.

## Background

Despite recent progress in health provision in many low- and middle-income countries (LMICs), the capacity of their health systems remains severely depleted. There is persistent exclusion and lack of access to basic quality services for large sectors of the population. It is well known that there are imbalances in access to essential services among regions and countries, between the urban, rural, and hard-to-reach areas, inadequate health infrastructures across countries, and shortages of human health resources [1]. In addition, such disparities follow economic lines, with access being at its lowest in low-income countries and in the most vulnerable populations. In these areas and populations, many people have insufficient money to pay for vital services or for transportation to reach them, or they are unable to take time away from their families, work, or school to use these services, even when they are available [2]. In addition, many worthy causes compete for limited public resources in LMICs, such that setting priorities for health is of special relevance to maximize the impact of investments.

The economic and social consequences of poor health service coverage and outcomes on families and communities are significant. Relevant social benefits can be gained by addressing the most vulnerable groups, such as children and mothers. For example, political will, as well as the prioritization of maternal health in budgeting, has driven progress in addressing maternal and newborn health issues in some African countries, such as Benin, Cameroon, and Uganda [3]. In this regard, attention must be paid to the fact that crucial health-care interventions, such as attending antenatal care and having a skilled health worker at the time of delivery, can generally prevent women from dying of pregnancy-related causes in developing countries [4]. Health issues are also intricately connected to the economic and developmental prospects of women and girls [5]; such issues are central to progress in global development efforts [6]. In this regard, the ancillary health, economic, and development benefits of family programs would be substantial. Moreover, reducing unmet family planning needs has unequivocally saved lives and money [1, 7]. The promotion of family planning in LMICs has the potential to reduce poverty and hunger and to prevent 30% of all maternal deaths and nearly 10% of childhood deaths [8]. Moreover, the additional immunisation costs decrease over time as population size decreases [9]. The United Nations [10] has estimated that, for “every dollar spent in family planning, between two and six dollars can be saved in interventions aimed at achieving other development goals”.

In this context, the Third United Nations (UN) Financing for Development Conference adopted the Addis Ababa Action Agenda (AAAA). Although this agenda involves boosting the financing of Sustainable Development Goals (SDGs), in many LMICs weak health systems are functioning with meagre and inequitably distributed resources [11]. This situation has arisen mainly because these countries have limited access to international financial markets, are particularly vulnerable to macroeconomic shocks, and frequently rely on volatile external financing sources, such as official development assistance (ODA). Thus, in addition to support from the international community, domestic resource mobilisation is the only sustainable strategy available to LMICs to finance their basic healthcare services [12]. However, substantial public revenues are lost due to the existence of illicit financial flows (IFFs), particularly in LMICs. These financial flows are cross-border transfers of funds that are illegally earned, transferred, or utilized. They are the main driving force behind the net drain of domestic financial resources from most countries in developing areas. Furthermore, these IFFs from developing countries steadily increased to reach US$ 1.1 trillion in 2013, exceeding the combined total ODA and foreign direct investment (FDI) received by these economies [13]. For example, to mention two very different and relevant cases, Togo lost approximately 34% of its GDP per year in the period 2008–2013 through illicit financial outflows, while Costa Rica lost an amount equivalent to 26% of its GDP. Moreover, in the case of Togo, the amount of resources lost through IFFs represents, on average, five times the total tax revenues in the period. In Costa Rica, the amount of IFFs was 2.8 times higher than the tax revenue managed by the central government within this period. Following Zucman [14], if we assume a rate of return of 5% for undeclared wealth and apply an average tax rate on investment incomes of 50% in the case of Togo and 58% in the case of Costa Rica (i.e. the average commercial tax rates in these countries according to the World Bank), the annual government’s revenue losses due to IFFs are estimated to be 12 and 8% of total public revenues, respectively. Thus, these figures show both the enormous magnitude of IFFs in these countries and their potential damaging impact on the capacity of public budgets. In this regard, the case of Africa is noteworthy. According to the African Development Bank and Global Financial Integrity (GFI) [15], Africa lost between US$ 1.2–1.3 trillion dollars over the period 1980–2009 through IFFs, an amount which is about four times Africa’s total external debt. Indeed, in 2009, IFFs out of Africa were more than three times the amount of ODA received. As a global response to this problem, the World Bank has established analytical and operational approaches to strategically address IFFs as a core developmental issue [16].

Thus, although the magnitude of IFFs and their potential role in damaging the capacity of the state budget in LMICs is increasingly recognized, a few studies have found evidence for the effect of IFFs on the provision of public goods and services [17–19]. These studies are examples of the few research efforts that have attempted to provide quantitative studies that assess the relationships between IFFs and health services. On the one hand, O’Hare et al. [17] found that IFFs had a significant effect on achieving the fourth target of the millennium development goal (MDG) (i.e. to reduce the under-five mortality rate by two-thirds between 1990 and 2015) in Sub-Saharan Africa (SSA) countries. In addition, O’Hare et al. [18] provided evidence on the time needed for the SSA region countries to reach this target if national resources had been fully mobilized (i.e. in the absence of leaks, such as IFFs, corruption, and debt service). On the other hand, the negative effect of IFFs on infant immunization coverage rates in LMICs was analysed by Ortega et al. [19].

Given this background, the main aim of this article was to analyse the association between cross-country differences in the level of IFFs and current dissimilarities in the coverage of basic health services across countries. In this sense, this study contributes to the existing literature [17–19] in several ways. Firstly, it provides evidence on the negative association between the increase in IFFs and the provision of a representative set of essential health services, including those related to the health care of vulnerable populations (i.e. child and maternal health). Secondly, this study also shows that the potential negative effect of IFFs is not uniformly distributed across regions and health services. In particular, these negative associations were detected in regions such as SSA, the Middle East, and North Africa (MENA), and countries included in the Europe & Central Asia (ECA) region.

The following section presents the analytical framework used and reviews the existing literature on the effects of IFFs on health development. Next, the data and the statistical methodology used in the empirical analysis are presented. Finally, the research findings are discussed and some conclusions are offered.

## Theoretical framework and related literature

A strong revenue generating capacity is fundamental for a developing country to sustainably generate financing for its own development [20–22]. However, the use of public funds from domestic sources to finance health has stagnated in most LMICs, without evidence that domestic public financing has been replaced by private financing [23]. For this reason, the SDGs and the AAAA recognize the importance of mobilising domestic revenue to ensure the financing of the 2030 Agenda for Sustainable Development. Indeed, SDG Target 17.1 is to “strengthen domestic resource mobilization, including through international support to developing countries, to improve domestic capacity for tax and other revenue collection”. Nevertheless, there is intense debate on the way to finance progress towards the health-related SDGs in LMICs. In many poor countries, the existence of a large informal sector (i.e., the part of the economy which escapes tax coverage) significantly reduces the governments’ capacity to raise public revenues above the critical threshold of 15% of GDP, below which threshold countries have difficulty in funding basic state functions [16]. In addition, these difficulties are often aggravated during domestic resource collection processes. Poverty may increase if, for example, these resources are collected by increasing consumption taxes — which is the most likely source to be effective [24] — but without implementing specific policies to improve the situation of the poor. Thus, one of the greatest risks in setting an ambitious domestic resource mobilization agenda is that in the process governments will impoverish poor people even further [25]. In addition, international corporate tax competition and trade liberalisation have also diminished the states’ capacity for domestic resource mobilisation [24].

On these grounds, the existence of IFFs is an issue that has received special attention within the policy coherence framework [26–28]. This issue has also been highlighted in the SDG 16.4: “By 2030, significantly reduce illicit financial and arms flows, strengthen the recovery and return of stolen assets, and combat all forms of organized crime” [29]. In fact, the WHO [30] stated that a country’s capacity to raise and spend funds domestically will be further enhanced if the statements of intent made in Addis Ababa to make tax systems more efficient nationally are realized by implementing effective measures to reduce tax evasion and illicit tax flows globally. In this regard, it is important to recall that Ndikumana et al. [31] suggested that each additional dollar allocated to debt servicing means 29 cents less allocated to public health spending. O’Hare and Curtis [32] also studied deficiencies in healthcare financing in Malawi, showing that these financial shortages could be filled by corporate tax incentives and tax revenues foregone due to IFFs. Similarly, Mosselmans [33] showed that, if current spending patterns were maintained, the lives of 1.9 million children could be saved every year if the resources lost due to tax evasion were available to governments. These facts are in line with recent trends in global health financing that show that growth in public health spending is largely a consequence of economic growth and fiscal expansion rather than a consequence of governments giving priority to health [34]. In addition, it is important to note that the growing relevance of IFFs from developing countries is a mirror of severe institutional and governance difficulties. There is growing evidence that IFFs widen inequalities in multiple ways, help to preserve unequal power relationships, and further weaken already weak state institutions [35].

**Fig. 1:**
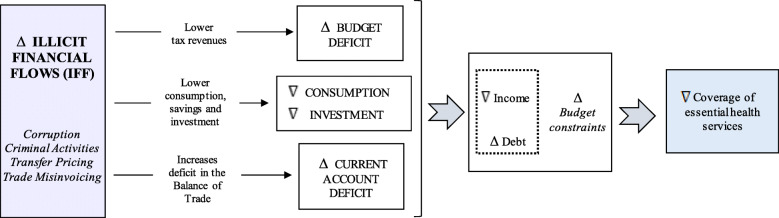
How IFFs can reduce the provision of health services

As shown in Fig. 1, IFFs may arise from a wide set of activities, such as corruption, criminal activities, transfer pricing, and trade misinvoicing. Although estimates of IFFs show that this phenomenon is widespread and highly persistent, people in developing countries are particularly hard hit, given that the quality (and scope) of the provision of public services could radically depend on the reduction of these illicit flows [36]. This is even more evident in environments in which international corporate tax competition has decreased the states’ capacity for domestic resource mobilization (‘races to the bottom’). For example, ‘if the tax breaks had not been granted and the health budget increased by 10-fold, the effect of Ebola on Sierra Leone might have been avoided’ [37].

From a broader perspective, IFFs lead to decreases in domestic resources, which influence consumption and reduce national savings and investment. As result of these losses of resources and tax revenues, as well as pressures exerted on the budget, a second channel of IFFs arises in the form of budget deficits, which lead to less social spending (health, education, and infrastructure) for socially and economically disadvantaged population. Furthermore, in many developing countries, increased IFFs are accompanied by increased foreign borrowing (the so-called ‘revolving door’ syndrome) [31, 38–44]. The commitment to pay off this debt and the debt service charges reduce the capacity of governments to increase social spending. Thus, the relationship between debt and illicit flows forms a vicious cycle; as countries borrow more, capital flight accelerates as corrupt politicians and officials divert loans offshore. Thus, the existence of IFFs leads to external flow imbalances in the economy, reduces foreign reserves, and increases current account deficits. Furthermore, IFFs not only reduce countries’ fiscal space but also promote inefficient public expenditures, thus increasing the cost and reducing the quality of the provision of public services.

Having drawn attention to these issues, it is relevant to note that the availability of additional government revenues has not always led to a commensurate increase in public investments in basic health services, as occurs in the case of aid fungibility for health in developing countries. This non-additionality of aid occurs when an increase in donor funding is associated with a proportionate decrease in government spending in health [45]. For example, ODA might relax the recipient government’s budget constraint and eventually lose public resources that can be spent on health services, but also on other social or non-social expenditures. The process by which these extra resources may be displaced from basic health services to other priorities may occur at two stages throughout the budget allocation process: firstly, during negotiations between different ministries for resources from the general budget; and, secondly, during the health sector allocation of its own funds [46]. On the one hand, the final allocation of these resources might depend on both the bargaining power of the different ministries and the prevailing budgeting priorities during this process. In this regard, it is important to point out that these priorities may change during this process (e.g. because of unexpected changes in the country’s macroeconomic and/or social context). This is especially evident in the case of fiscally constrained governments with a wide range of priorities in which these extra-resources may be invested. On the other hand, when it can be assumed that these extra resources will be fully invested in health, this does not imply that the entire additional funding will be invested on basic services. As a result of the availability of such additional financing, there could be circumstances in which government health priorities could shift from basic health services to secondary or even tertiary services. In addition, it can be assumed that the potential effect of the availability of additional resources on the provision of basic health services may be direct and/or induced or indirect. Regarding the indirect effect, it is important to note that government investments in other competing priorities may also have positive effects on health achievements. Such competing priorities may include education, especially in the case of female education, and some kinds of infrastructure investments (e.g. those related to water provision).

Finally, it is important to note that the main illicit capital flight comes from trade misinvoicing [47]. Trade misinvoicing is a form of customs and/or tax fraud involving exporters and importers deliberately misreporting the value, quantity, or nature of goods or services in a commercial transaction [48]. This issue has received increasing attention through the debates on IFFs, because trade misinvoicing continues to be used as a key mechanism to fuel IFFs from developing countries [49]. In fact, of the total annual illicit financial outflows in LMICs, close to 80% of the funds are moved offshore using trade misinvoicing [47]. This problem also motivated the UN Economic Commission for Africa (UNECA) and the African Union to establish a High Level Panel on Illicit Financial Flows from Africa. This panel estimated that trade misinvoicing, which is concentrated on a few products, is the reason for $50 billion worth of illicit flows from Africa [50]. These estimated high levels of trade misinvoicing have played a key role in the decision to incorporate the issue of IFFs into the Sustainable Development Goals [51].

## Methods

### Data

The original database comprised data from 73 countries. However, no data were available on the variable of interest (IFF/Trade) for Sao Tome and Principe. For this reason, this country was not included in the regression analyses, although data exists for the dependent variables. Thus, this study used data for the available sample of 72 LMICs for the period 2008 to 2013. However, due to data availability constraints, a panel could not be constructed for all variables, as can be seen in Table A1 (see Additional file 1). This table provides information on the annual observations available for each country included in the original database. The table shows that there are three dependent variables (*Antenatal Care*, *Hospital Beds*, and *Physicians*) for which only one or two annual observations are available for most countries, and which do not always correspond to the same year within the period 2008–2013. For example, in the specific case of ‘Antenatal care’, 2010 was the year in which more cross-section observations were available within this period. For 2010, we only have information for 23 countries in the sample. Furthermore, in the case of 2008, we only have information for 12 countries in the sample. In addition, in most cases (47 countries), we have a point-in-time observation available within the period 2008–2013. For these reasons, the original data was transformed in order to obtain representative cross-sectional data points for the period. These cross-sectional data was obtained by ‘period-averaging’ the available data points in the sample. Thus, when a variable had more than one data point within the period in a country, the arithmetic mean of the available temporal observations for this variable was calculated to obtain a representative value for this country. However, for certain countries and variables, if just one temporal observation was available within the period (one point in time), it was considered to be the representative value for the entire period [52]. The literature shows that there could be additional reasons for using the ‘period averaging method’ when employing cross-section time series datasets. For example, when temporal data are very scattered across countries (with a profusion of missing data points), are potentially subject to measurement errors (especially when errors are non-random), and their levels do not fluctuate very much within the period considered (as is our case), then cross-sectional regressions based on period averages may be more effective than more sophisticated statistical models which employ pooled data or available full time series information [53]. Thus, the variables used, data imputation, country selection, and statistical approach employed were strongly constrained by data availability. Tables 1 and 2 show the variables included in the database and the list of countries, respectively.

**Table 1:** Variables employed in the estimations

Variable	Definition	Data source
*Family planning, coverage*	The percentage of total demand for family planning among married or in-union women aged 15–49 years	World Health Organization
*Antenatal care, coverage*	Percentage of women attended at least once during pregnancy by skilled health personnel for reasons related to pregnancy.	World Bank
*DTP3, coverage*	The percentage of 1-year-olds who have received three doses of combined diphtheria, tetanus toxoid and pertussis vaccine in a given year	World Health Organization
*Measles, coverage*	The percentage of children less than 1 year who have received at least one dose of measles-containing vaccine in a given year	World Health Organization
*Tuberculosis, coverage*	Tuberculosis effective treatment = Case detection rate (all forms) · Treatment success rate for all new cases	World Health Organization
*Sanitation, Coverage*	Access to improved sanitation facilities refers to the percentage of the population with at least adequate access to excreta disposal facilities ranging from simple but protected pit latrines to flush toilets with a sewerage connection	World Health Organization
*Hospital beds*	Total number of beds per 1000 population. Hospital beds include inpatient beds available in public, private, general, and specialized hospitals and rehabilitation centres	World Health Organization
*Physicians*	Total number of physicians per 1000 population	World Health Organization
*IFF*	IFFS are illegal movements of money or capital in current US$ from one country to another. The illegal capital outflows stem from crime, corruption, tax evasion, and other illicit activity. Current US $.	Global Financial Integrity
*Trade*	Total trade, in current US$	World Trade Organization
*IFFT*	(IFF / Trade) · 100	Authors
*GDPpc*	GDP per capita, in constant 2011 international $	World Bank
*GIR, 1st grade of primary education, female*	Gross intake ratio in first grade of primary education is the number of new entrants in the first grade of primary education regardless of age, expressed as a percentage of the population of the official primary entrance age	United Nations
*Urban population*	Percentage of people living in urban areas, as defined by national statistical offices	United Nations
*Births attended*	Births attended by skilled health staff (% of total)	World Bank
*CPI*	Corruption Perceptions Index, values from 0 (highly corrupt) to 10 (clean country)	Transparency International
*Density*	Population density (people per km^2^)	United Nations
*Dependency*	Dependency ratio is the ratio (in %) of dependents (people younger than 15 or older than 64 years) to the working-age population (those aged 15–64 years).	World Bank
*Literacy*	Adult literacy rate is the percentage of people aged 15 years or more who can both read and write with understanding a short simple statement about their everyday life.	United Nations
*Labour force participation rate, female*	The female labour force as a percentage of the total shows the extent to which women are active in the labour force. Labour force comprises people aged 15 years or more who supply labour for the production of goods and services during a specified period	World Bank
*Population*	Total population between the ages 15 to 64 as a percentage of the total population. Population is based on the de facto definition of population, which counts all residents regardless of legal status or citizenship.	United Nations
*EAP*	EAP equals 1 for the countries that belong to the East Asia & Pacific region, and equals 0 otherwise.	Authors
*ECA*	ECA equals 1 for the countries that belong to the Europe & Central Asia region, and equals 0 otherwise.	Authors
*LAC*	LAC equals 1 for the countries that belong to the Latin America & Caribbean region, and equals 0 otherwise.	Authors
*MENA*	MENA equals 1 for the countries that belong to the Middle East & North Africa region, and equals 0 otherwise.	Authors
*SA*	SA equals 1 for the countries that belong to the South Asia region, and equals 0 otherwise.	Authors
*SSA*	SSA equals 1 for the countries that belong to the Sub-Saharan Africa region, and equals 0 otherwise.	Authors

**Table 2:** Low- and middle-income countries in the sample by region

*Sub-Saharan Africa* (SSA)	*Latin America & Caribbean* (LAC)	*Europe & Central Asia* (ECA)	*East Asia & Pacific* (EAP)	*Middle East & North Africa* (MENA)	*South Asia* (SA)
Botswana	Argentina	Albania	Cambodia	Algeria	Bangladesh
Burkina Faso	Belize	Armenia	China	Djibouti	India
Cabo Verde	Brazil	Azerbaijan	Indonesia	Jordan	Maldives
Cameroon	Colombia	Belarus	Lao PDR	Morocco	
Chad	Costa Rica	Bulgaria	Malaysia	Tunisia	
Congo, Rep.	Dominican Rep.	Croatia	Philippines		
Cote d’Ivoire	Ecuador	Georgia	Thailand		
Ethiopia	El Salvador	Kazakhstan	Vietnam		
Gambia, The	Guatemala	Moldova			
Guinea-Bissau	Guyana	Romania			
Lesotho	Haiti	Russian Fed.			
Madagascar	Honduras	Serbia			
Malawi	Jamaica	Ukraine			
Mali	Mexico				
Namibia	Nicaragua				
Niger	Panama				
Sao Tome and Principe	Paraguay				
Sierra Leone	Peru				
South Africa	Suriname				
Sudan	Venezuela, RB				
Swaziland					
Tanzania					
Togo					
Zambia					

The key variable used in this study was the ratio of IFFs to total trade in each country. Given that, by construction, the volume of IFFs in a country is strongly restricted by the quantity of financial flows originating from trade [54], the indicator of the relative importance of the IFFs used was calculated as a ratio of the IFFs to total trade (IFFT). On the other hand, a normalisation of IFFs in terms of GDP or population may give the misleading impression that, for large countries with relative high GDP and large population, the magnitude of IFFs is not a relevant issue. This is the case, for example, of Nigeria. It is well known that this country has a severe illicit financial outflow problem, but this country ranks low in the list of IFF/population ratios due to its large population [15 , p. 27]. Another example is provided by the high levels of IFF/GDP in Vietnam (2.70%) compared to Indonesia (0.90%) in the period 2008–2013 (i.e. three times greater in the case of Vietnam). This ratio was a direct consequence of differences in the size of the GDP between these economies (i.e. the GDP of Indonesia is 5.3 times higher than that of Vietnam). However, these results do not reflect differences in the relevance of IFFs in these economies. For these reasons, it was considered that IFFT (i.e. how much money was transferred illicitly to foreign countries for each dollar traded), rather than IFF/GDP or IFF per capita, was an unbiased measure of the scale of this problem. Thus, the variable IFFT was obtained following the methods described in Ortega et al. [19], in which annual panel IFFT data for 56 low- and middle-income countries for the period 2002–13 were calculated.

**Fig. 2:**
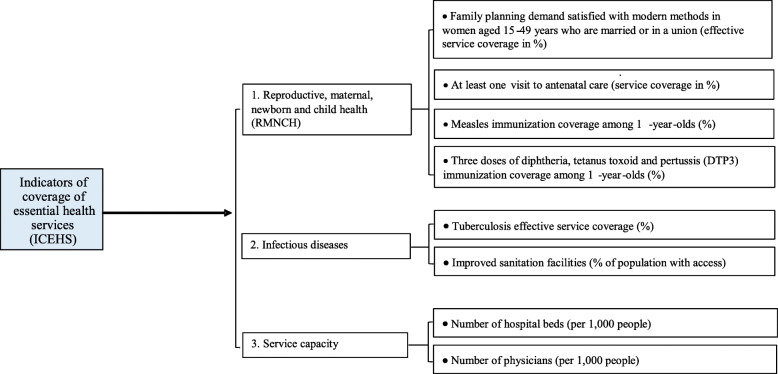
Selected essential health services indicators

In addition, key health activity coverage is a widely accepted indicator of progress toward improving health systems and achieving SDGs. Thus, following the proposal by Hogan et al. [55], a set of indicators that represent overall essential health services coverage was selected for analysis. These indicators included the following areas: reproductive, maternal, newborn, and child health; infectious diseases; non-communicable diseases; and service capacity. However, indicators related to the prevention and management of non-communicable diseases were excluded from the analysis due to the widespread scarcity of data. Figure 2 shows the final indicators selected, which are classified by category.

### Empirical model and estimation strategy

The following cross-sectional model was used as a baseline specification to analyse the association between the relative size of IFFs and the selected indicators representing overall coverage of essential health services (indicators of coverage of essential health services, ICEHS):

1 $$ {\mathrm{ICEHS}}_{\mathrm{ji}}=\upalpha\ {\mathrm{lagIFFT}}_{\mathrm{i}}+\upbeta\ \ln\ {\mathrm{GDPpc}}_{\mathrm{i}}+{\upvarepsilon}_{\mathrm{i}} $$

where i = 1, …, N are the 72 countries in the available sample. ICEHS_ji_ represents the imputed values for the period 2008–2013 of the specific indicator (j) for country (i) (see Table 3) and ε_i_ represents the error term. The variable (lagIFFT) represents mean values of the variable IFFT for the period 2002–2007 as it was assumed that it takes time before a change in the relative magnitude of IFFs from a country may have an effect on the provision of essential health services. For this reason, this variable was lagged by one period, such that the IFFT in period 2002–2007 had an effect on health service indicators in 2008–2013. Eq. (1) also includes as an independent variable the average level of GDP per capita in natural logs (ln GDPpc) for the period 2008–2013, which in general is a proxy for overall affluence in a country. This factor may affect ICEHS in ways not mediated by other determinants of the provision of essential services.

**Table 3:** Descriptive analysis of the variables used in the estimations

Variables	Obs.	Mean	SD.	Min.	Max.
Dependent					
*Family planning, coverage (%)*	73	59.94	20.51	14.63	95.32
*Antenatal care, coverage (%)*	65	90.45	11.93	42.50	100.00
*DTP3, coverage (%)*	73	88.13	10.86	34.67	99.00
*Measles, coverage (%)*	73	87.67	11.27	47.67	99.00
*Tuberculosis, coverage (%)*	73	52.88	15.12	25.15	79.58
*Sanitation, coverage (%)*	73	62.67	28.51	9.63	98.48
*Hospitals beds (per 1000 people)*	64	2.43	2.20	0.20	11.20
*Physicians (per 1000 people)*	66	1.18	1.21	0.02	4.50
Independent					
*Lag (IFFT) (%)*	72	12.87	10.72	1.07	49.09
*GDPpc (constant 2011 international $* per capita*)*	73	8193.31	5872.03	830.51	23,888.91
*GIR, 1st grade of primary education, female (%)*	68	106.7	19.70	63.37	178.33
*Urban population (% of total)*	73	52.32	18.62	15.62	91.04
*Lag (IFFT) · EAP*	72	0.82	2.51	0	10.43
*Lag (IFFT) · ECA*	72	2.47	6.63	0	37.47
*Lag (IFFT) · LAC*	72	3.88	8.64	0	36.27
*Lag (IFFT) · MENA*	72	0.73	4.60	0	38.47
*Lag (IFFT) · SA*	72	0.29	1.59	0	11.87
*Lag (IFFT) · SSA*	72	4.68	9.44	0	49.09
Excluded instruments					
*Births attended (% of total)*	72	80.06	23.04	16.55	99.9
*CPI*	73	3.26	0.83	1.79	6.03
*Density (people per km*^*2*^*)*	73	117.56	203.42	2.70	1246.67
*Dependency (% of total population)*	73	37.97	6.79	26.40	52.50
*IFF/GDP (%)*	73	4.84	5.47	0.33	34.00
*Labour force participation rate, female (%)*	73	54.08	15.50	14.67	88.27
*Lag (Births attended)(%)*	73	73.74	27.27	6.05	99.9
*Lag (CPI)*	73	3.03	0.86	1.62	5.83
*Lag (GDPpc) (constant 2011 international $* per capita*)*	73	6666.92	4788.92	718.43	19,126.04
*Lag (IFF/GDP)(%)*	73	3.62	3.47	0.23	15.49
*Lag (IFF/Population) (international $* per capita*)*	73	234.90	266.99	4.15	1150.28
*Lag (IFF/Population) · EAP*	73	22.65	135.85	0	1150.28
*Lag (IFF/Population) · ECA*	73	66.65	180.48	0	808.17
*Lag (IFF/Population) · LAC*	73	91.16	217.67	0	1101.87
*Lag (IFF/Population) · MENA*	73	9.56	45.71	0	344.27
*Lag (IFF/Population) · SA*	73	4.02	30.08	0	256.19
*Lag (IFF/Population) · SSA*	73	40.86	102.81	0	469.57
*Lag (Labour force participation rate, female)(%)*	73	53.09	15.48	12.67	88.23
*Lag (Urban population)(%)*	73	49.66	18.84	15.01	89.99
*Literacy*	60	81.53	19.98	15.46	99.97
*Ln Population (inhabitants)*	73	16.24	1.77	12.08	21.02

Averages for the period 2008–2013. Lagged values of variables refer to averages of variables for the previous period 2002–2007

Finally, for the sake of simplicity and comparability between different models, an augmented model was estimated using the additional controls. (X_i_) represents a set of (k) variables (also mean values for the period 2008–2013) which themselves may not be considered a direct outcome of the level of IFFTs.

2 $$ {\mathrm{ICEHS}}_{\mathrm{ji}}=\upalpha\ {\mathrm{lagIFFT}}_{\mathrm{i}}+\upbeta\ \ln\ {\mathrm{GDPpc}}_{\mathrm{i}}+{\upgamma}_{\mathrm{k}}{\mathrm{X}}_{\mathrm{k}\mathrm{i}}+{\upvarepsilon}_{\mathrm{i}} $$

These control variables are as follows (see Table 3 and Table A2 in additional file 1):

- *Gross intake ratio in 1st grade of primary education*, female, which was used as a proxy for the educational level of mothers in the country. The use of this proxy was based on the assumption that educated mothers are more likely to adopt preventive behaviours and to use maternal and infant health services.
- *Urban population, as a percentage of the total population*. This variable was chosen on the basis that rural populations lag behind their urban counterparts in access to most of the essential health services [56].

It important to note that the literature shows that there are additional factors not included in eq. (2) that have an impact on the coverage of essential health services (e.g. public and private health expenditures, donor assistance to health, or the quality of governance). However, given that these variables could themselves be considered outcomes of the variable of interest (IFFT) (see [31] –[36]), these additional controls were not included in eq. (2) with the aim of avoiding a potential ‘bad control’ problem [57].

In eqs. (1) and (2), the parameter of interest is (α). This coefficient quantifies the strength of association between lagIFFT and the different indicators included in variable ICEHS, all other control variables being equal. For the reasons previously discussed, it was expected that the estimated coefficient (α) would be negative and significant in both models. This result would show that a higher level of IFFT in the period 2002 to 2007 would be associated with a reduction in ICEHS coverage over the next period 2008 to 2013.

The classic linear regression model includes a wide range of circumstances in which the explanatory variables are endogenous (i.e. are correlated with the disturbance), such that OLS regression does not yield consistent estimates. In fact, a regression model can have more than one source of endogeneity. The empirical model proposed in this article includes at least three relevant examples of these circumstances: measurement errors in variables, relevant omitted variables, and simultaneity bias due to reverse causality. As a way to illustrate the endogeneity problem in the estimation of baseline model, it should be recalled that we are assuming that this model allows us to quantify what effect (if any) a change in the “independent” lagIFFT might have on the “dependent” variable (e.g. DTP3), all else being equal. On the one hand, the observed existence of a negative relationship between IFFT and DTP3 levels could be the consequence of the existence of an unobserved effect generated by an omitted variable, such as the country’s corruption level. This would have an effect on both variables (i.e. an increase in corruption may decrease DTP3 coverage levels and, at the same time, increase the level of IFFT). Thus, both DTP3 and IFFT move in opposite directions because both variables are impacted by a change in the level of corruption rather than by the existence of a direct causal relationship between both variables. On the other hand, it is relevant to take into account that eq. (1) defines a causal ordering between the variables: the value of DTP3 follows from those of IFFT and this implies that IFFT should not be affected by changes in DTP3 (i.e. IFFT should be exogenous). Otherwise, the existence of inverse causality (or simultaneity) would lead to the OLS estimator giving biased and inconsistent estimates of parameters (i.e. simultaneity bias). One way to avoid these problems would be to employ instrumental variable (IV) methods, which generate consistent estimates provided that it is possible to find instruments that are (at least asymptotically) correlated with the endogenous independent variable and uncorrelated with the error term.

Sometimes theory can suggest which explanatory or independent variables in a model may be potentially endogenous, especially in the case of simultaneity or omitted variables, but it cannot indicate whether the correlation of regressors with the error term is sufficiently large as to invalidate the OLS estimation due to its inconsistency. One solution to this problem is to study the possible endogeneity of the regressors from an empirical perspective and perform tests to determine whether the independent variables being tested have to be treated as endogenous. In applied research, an independent variable is said to be endogenous if it is correlated with the error term [58]. This forms the basis to study the possible endogeneity of the regressors from an empirical perspective independently of the exact source of endogeneity. For this reason, we employed endogeneity tests to study whether independent variables can be treated as exogenous ones.

However, prior to this approach, instruments have to be identified that are strongly correlated with the potentially endogenous variables but are uncorrelated with the error term. Regarding this point, it is crucial to ensure the relevance of the instruments, because if the instruments used are weak, endogeneity tests can lead to misleading results. In other words, instrumentation is made possible by a set of instrumental variables that fulfil the following conditions: they are uncorrelated with the disturbance term (are exogenous); they are correlated with the endogenous regressor (are relevant); and they are not included in the original equation (the instruments have no causal impact on the dependent variable except through their effects on the endogenous variable). Thus, a relevant and exogenous instrument may capture the part of the variations of the endogenous variable which are exogenous. These exogenous variations can also be used to estimate the truth effect of the endogenous variable on the dependent variable by means of IV estimators (such as the Two-Step Least Squares [2SLS] estimator).

In this study, the endogenous regressors and the excluded instruments considered in each regression are identified in the footnotes to the tables showing the estimations results. In order to test whether a regressor can be treated as exogenous, we used the endogtest()option of the ivreg2 program developed in Stata [59]. This option reports a test statistic that is robust to various violations of conditional homoscedasticity. If the results of these tests applied to the explanatory variables in the model show that the hypothesis of exogeneity cannot be rejected, then, in principle, these variables can themselves be valid (included) instruments for the estimation. In those cases where the exogeneity hypothesis can be rejected, excluded instruments were used for the endogenous regressors in the model. Thus, in order to test whether the excluded instruments were relevant, we used an underidentification test (heteroscedasticity-robust Kleinbergen-Paap rk LM statistic) and a weak identification test (the robust Kleinbergen-Paap rk Wald F statistic). We also used a test for redundancy of instruments and the robust Hansen’s J statistic to select a set of excluded instruments which are uncorrelated with the error term and are correctly excluded from the estimated equation. An additional file shows this procedure in more detail (see Additional file 2).

## Results

**Fig. 3:**
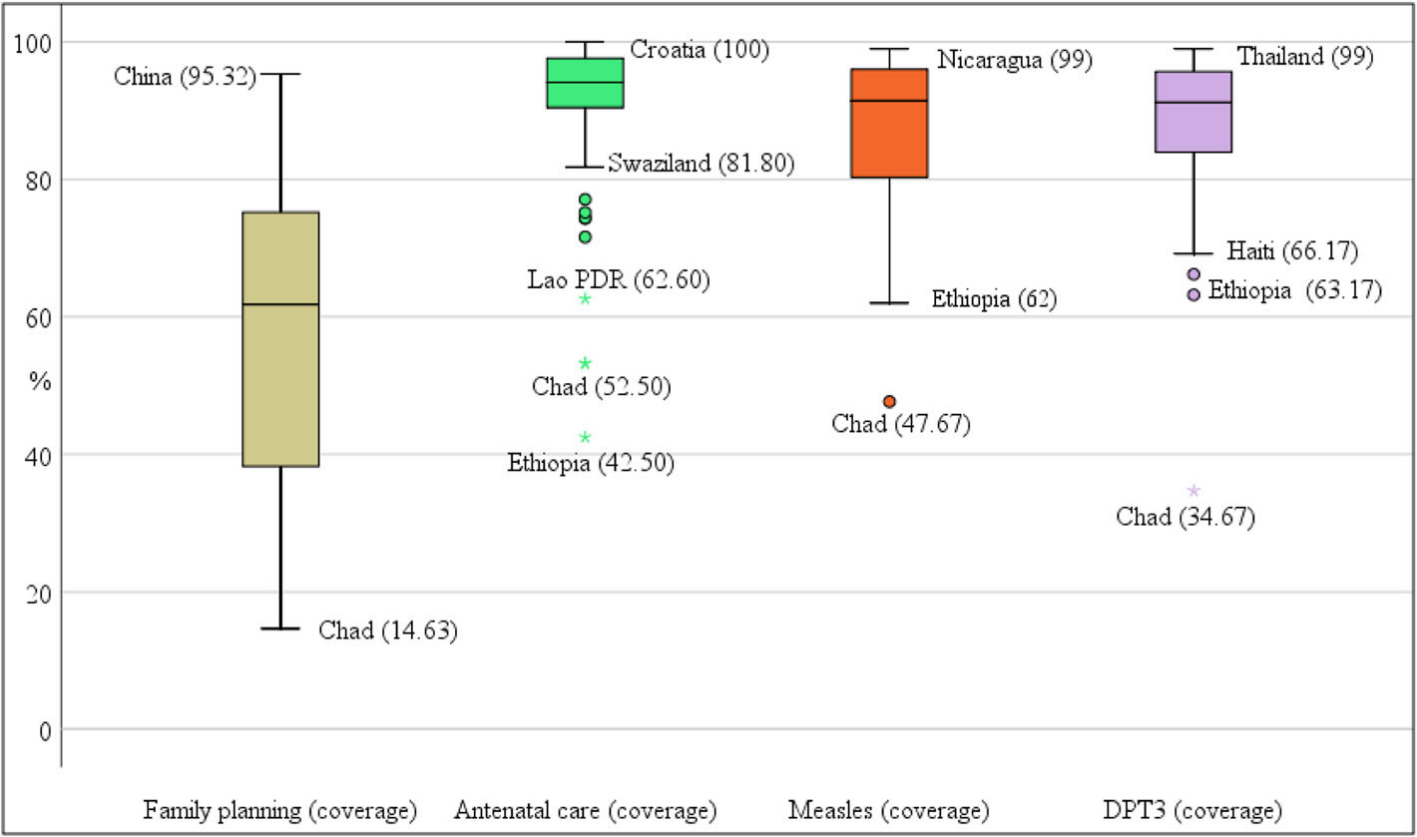
Box-and-Whisker plots of individual RMNCH tracer indicators for the countries in the sample and the period 2008 to 2013. The box in the plot represents the interquartile range. The top of the box demarcates the third quartile and the bottom denotes the first. The horizontal line in the middle of the box represents the median of the distribution. The circles and asterisks represent outliers. The horizontal lines (whiskers) indicate the maximum and minimum values of the distribution excluding outliers. Average values for the period are shown in parentheses

**Fig. 4:**
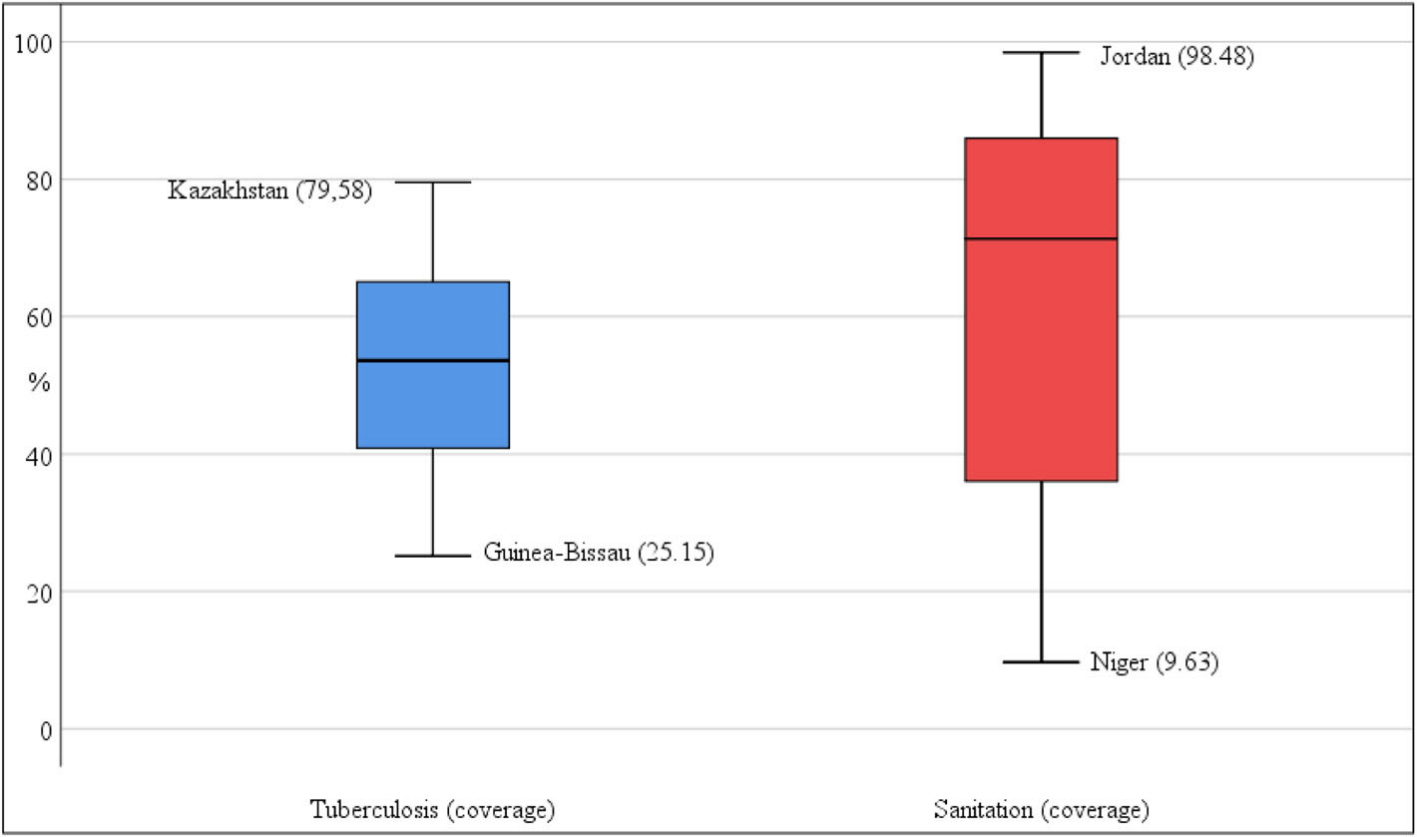
Box-and-Whisker plots of individual infectious diseases tracer indicators for the countries in the sample and the period 2008 to 2013. The box in the plot represents the interquartile range. The top of the box demarcates the third quartile and the bottom denotes the first. The horizontal line in the middle of the box represents the median of the distribution. The circles and asterisks represent outliers. The horizontal lines (whiskers) indicate the maximum and minimum values of the distribution excluding outliers. Average values for the period are shown in parentheses

**Fig. 5:**
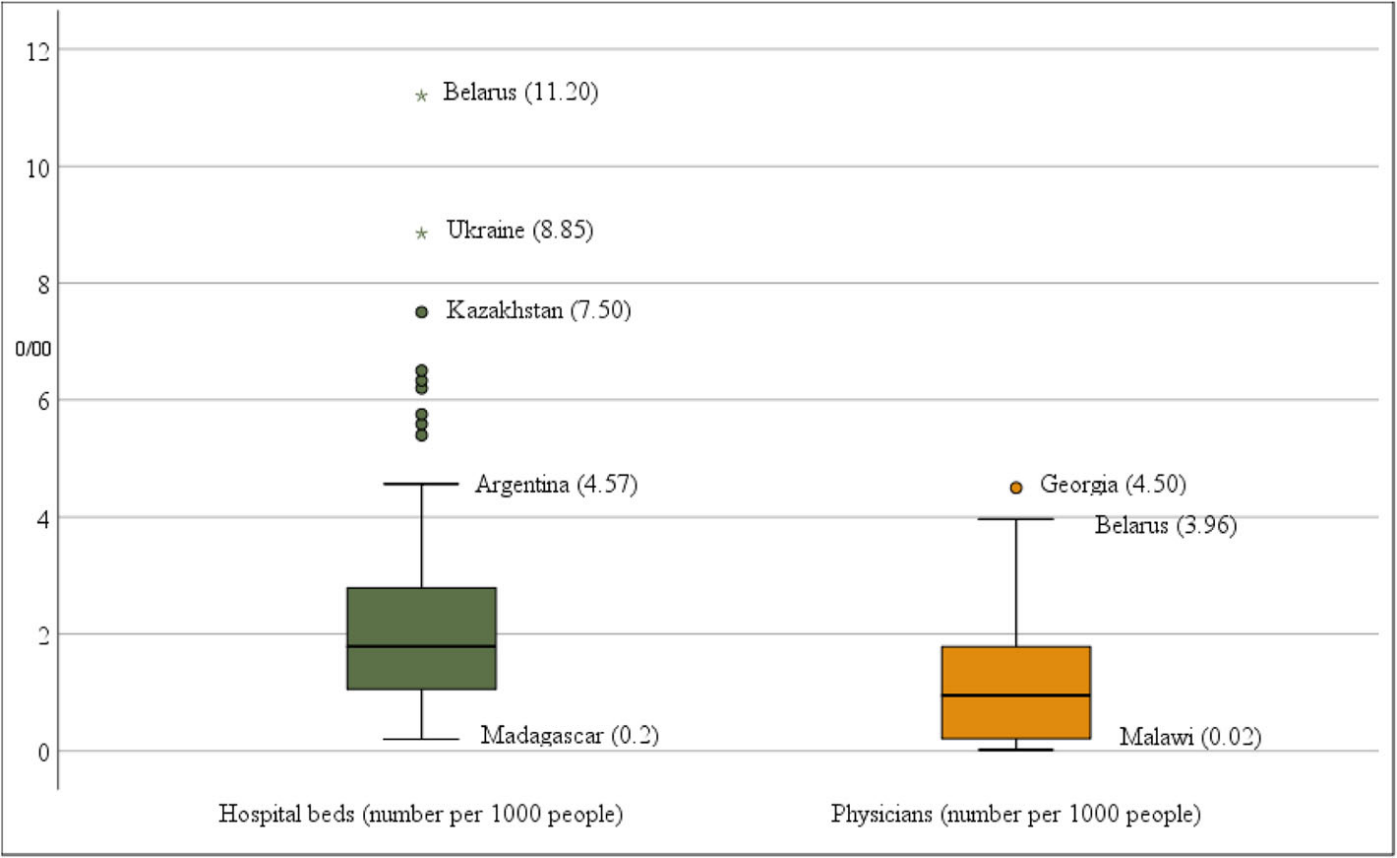
Box-and-Whisker plots of individual service capacity tracer indicators for the countries in the sample and the period 2008 to 2013. The box in the plot represents the interquartile range. The top of the box demarcates the third quartile and the bottom denotes the first. The horizontal line in the middle of the box represents the median of the distribution. The circles and asterisks represent outliers. The horizontal lines (whiskers) indicate the maximum and minimum values of the distribution excluding outliers. Average values for the period are shown in parentheses

Box-and-Whisker plots are shown in Figs. 3, 4, and 5, which depict the distribution of values across countries by individual tracer indicators. In the case of reproductive, maternal, newborn, and child health (RMNCH) indicators, Fig. 3 shows that the family planning coverage has the greatest dispersion, ranging from 15% (Chad) to 95% (China). Antenatal care coverage has the lowest interquartile range after excluding outliers such as Ethiopia, Chad, and Lao PDR. Child immunisation indicators (measles and DPT3 coverage) have very similar distributions, with a median value of around 88%. However, after excluding outliers, the distribution of measles immunisation coverage has a higher degree of asymmetry.

Figure 4 shows the plots of the two individual indicators for infectious diseases. There was also substantial variation in household access to at least basic sanitation coverage, ranging from 9.6% in the case of Niger to 98.5% in the case of Jordan. Regarding this public health key intervention, it can be seen that the interquartile range is even greater than that of family planning coverage. However, there were no outliers for tuberculosis and basic sanitation indicators. Finally, Fig. 5 shows the distribution of values of the service capacity indicators. Current values across countries for the indicator hospitals beds per 1000 people ranged from 0.2 (Madagascar) to 11.2 (Belarus), with a median value of less than 2 beds per 1000 people. However, Belarus, among other countries, is a clear outlier in this distribution. Excluding these outliers, the box shows that the maximum value of the distribution is 4.5 beds per 1000 people (corresponding to Argentina). The values for physicians per 1000 people ranged 0.02 (Malawi) to 4.5 (Georgia), and also have a very asymmetric distribution.

As mentioned, the 2SLS estimator was used to control for endogeneity and to eliminate biases and inconsistencies in the estimations for each ICEHS indicator. Table 4 shows the estimation results (heteroscedasticity-consistent) for eq. (1). In most cases, all the estimated coefficients presented the expected signs in the regressions. A negative association was found between the relative level of lagIFFT and all RMNCH indicators (Table 4, columns 1–4). No significant association was found in the case of infectious diseases indicators (effective tuberculosis treatment coverage and the coverage of households with access to basic sanitation), and the service capacity indicators considered (Table 4, columns 5–8).

**Table 4:** 2SLS estimations. Baseline model, eq. (1)

Independent variables	Dependent variable							
	*Family planning* (1)	*Antenatal care* (2)	*DTP3* (3)	*Measles* (4)	*Tuberculosis* (5)	*Sanitation* (6)	*ln Beds* (7)	*ln Physic* (8)
*Lag (IFFT)*	−0.299 (0.157)	−0.340^a^ (0.089)	− 0.611^c^ (0.007)	− 0.426^b^ (0.021)	0.106 (0.350)	− 0.254 (0.389)	− 0.001 (0.934)	−0.020 (0.278)
*ln GDPpc*	7.40^c^ (0.000)	10.96^c^ (0.000)	10.95^c^ (0.000)	10.66^c^ (0.000)	6.05^c^ (0.000)	7.81^c^ (0.000)	0.07^c^ (0.000)	−0.04 (0.287)
RESET test (*p*-value)	4.76 (0.033)	1.54 (0.219)	3.87 (0.053)	4.16 (0.045)	2.00 (0.163)	56.62 (0.000)	14.03 (0.000)	7.20 (0.009)
K-P rk LM (p-value)	46.70 (0.000)	11.08 (0.004)	12.30 (0.002)	12.30 (0.002)	36.65 (0.000)	14.18 (0.001)	11.90 (0.003)	12.08 (0.002)
K-P rk F (S-Y 10% max. IV size)	88,671.96 (19.93)	9.68 (13.43)	16.48 (19.93)	16.48 (19.93)	79,885.44 (19.93)	81.77 (19.93)	48.94 (13.43)	87.86 (19.93)
J statistic (p-value)	1.11 (0.291)	0.09 (0.764)	0.03 (0.850)	0.04 (0.835)	0.32 (0.569)	0.26 (0.608)	0.17 (0.685)	0.05 (0.817)
# Countries	72	64	72	72	59	72	63	66

Estimated coefficients and statistics, *p*-values in brackets. ^a^ Denotes coefficient statistically significant at the 10% level, ^b^ at the 5% level, and ^c^ at the 1% level. Standard errors and covariance are heteroscedasticity-consistent. *RESET* is the regression equation specification error (heteroscedastic-robust) test designed to test for missing (excluded) regressors. It also has great power to detect non-linearities in the model. Thus, rejection of the null hypothesis could be due to either a nonlinearity or an omitted explanatory variable. The model is identified if the heteroscedasticity-robust Kleinbergen-Paap rk LM statistic for *underidentification test* (*K-P rk LM)* rejects the null hypothesis. If the robust Kleinbergen-Paap rk Wald F statistic for *weak identification test (K-P rk F)* is greater than the Stock and Yogo [60] critical value (S-Y 10% max. IV relative size), then the null hypothesis of weak instruments can be rejected. The null hypothesis of the robust Hansen’s *J statistic* is that the instruments are uncorrelated with the error term and that excluded instruments are correctly excluded from the estimated equation. The excluded instruments used in the estimations for the instrumented variables are as follows (lagged values of variables (lag) refer to averages of variables for the period 2002–2007):

- Column (1). Instrumented: ln GDPpc. Excluded instruments: lag (Urban), lag (ln GDPpc)

- Column (2): Instrumented: lag (IFFT), ln GDPpc. Excluded instruments: lag (CPI), lag (IFF/Population), lag (ln GDPpc)

- Column (3): Instrumented: lag (IFFT). Excluded instruments: lag (CPI), lag (IFF/Population)

- Column (4): Instrumented: lag (IFFT). Excluded instruments: lag (CPI), lag (IFF/Population)

- Column (5): Instrumented: ln GDPpc. Excluded instruments: Literacy, lag (ln GDPpc)

- Column (6): Instrumented: lag (IFFT). Excluded instruments: lag (CPI), lag (IFF/GDP)

- Column (7): Instrumented: lag (IFFT), ln GDPpc. Excluded instruments: lag (CPI), lag (IFF/GDP), lag (ln GDPpc).

- Column (8): Instrumented: lag (IFFT). Excluded instruments: lag (CPI), lag (IFF/GDP)

Estimating eq. (2) (i.e. the baseline model with additional controls), the 2SLS results confirm that there was a negative association between IFFT and all RMNCH indicators, and that there were no significant associations between IFFT and infectious diseases and IFFT and service capacity indicators. The robustness of the results obtained using the 2SLS estimator is granted if we consider that no problems related to under-identification, weak instruments, or correlation between employed instruments and the disturbance terms were found in any of the estimations (Table 5).

**Table 5:** 2SLS estimations. Baseline model with additional controls, eq. (2)

Independent variables	Dependent variable							
	*Family planning* (1)	*Antenatal care* (2)	*DTP3* (3)	*Measles* (4)	*Tuberculosis* (5)	*Sanitation* (6)	*ln Beds* (7)	*ln Physic* (8)
*Lag (IFFT)*	−0.460^b^ (0.040)	−0.312^b^ (0.031)	− 0.307^b^ (0.017)	− 0.334^b^ (0.011)	0.113 (0.630)	0.193 (0.562)	0.002 (0.781)	0.007 (0.780)
*ln GDPpc*	−0.80 (0.775)	8.54^c^ (0.000)	8.50^c^ (0.000)	10.77^c^ (0.000)	5.25^c^ (0.000)	18.85^c^ (0.000)	0.18^b^ (0.025)	0.52^b^ (0.034)
*GIR female*	0.314^b^ (0.011)	0.134^b^ (0.044)	0.185^b^ (0.002)	0.051 (0.680)	− 0.016 (0.817)	− 0.982^c^ (0.000)	− 0.012^b^ (0.011)	− 0.060^c^ (0.002)
*Urban*	0.754^c^ (0.005)	0.122 (0.200)	−0.033 (0.750)	− 0.132 (0.214)	0.150 (0.160)	0.045 (0.872)	0.047 (0.499)	0.023 (0.129)
RESET test (*p*-value)	0.09 (0.770)	0.01 (0.942)	3.77 (0.057)	0.00 (0.984)	0.48 (0.492)	3.16 (0.081)	0.38 (0.542)	0.42 (0.518)
K-P rk LM (*p*-value)	14.91 (0.001)	15.11 (0.000)	16.18 (0.001)	26.00 (0.000)	14.85 (0.000)	12.49 (0.002)	23.62 (0.000)	12.51 (0.000)
K-P rk F (S-Y 10% max. IV size)	24.09 (19.93)	9.74 (19.45)	12.05 (16.87)	14.79 (16.87)	26.27 (19.93)	9.49 (13.43)	786.81 (13.43)	12.99 (19.93)
J statistic (*p*-value)	0.29 (0.592)	4.72 (0.193)	1.26 (0.534)	1.15 (0.563)	0.12 (0.735)	0.00 (0.997)	0.02 (0.894)	0.18 (0.670)
# Countries	67	60	67	67	67	66	60	62

Estimated coefficients and statistics, p-values in brackets. ^a^ Denotes coefficient statistically significant at the 10% level, ^b^ at the 5% level, and ^c^ at the 1% level. Standard errors and covariance are heteroscedasticity-consistent. *RESET* is the regression equation specification error (heteroscedastic-robust) test designed to test for missing (excluded) regressors. It also has great power to detect non-linearities in the model. Thus, rejection of the null hypothesis could be due to either a nonlinearity or an omitted explanatory variable. The model is identified if the heteroscedasticity-robust Kleinbergen-Paap rk LM statistic for *underidentification test* (*K-P rk LM)* rejects the null hypothesis. If the robust Kleinbergen-Paap rk Wald F statistic for *weak identification test (K-P rk F)* is greater than the Stock and Yogo [60] critical value (S-Y 10% max. IV size), then the null hypothesis of weak instruments can be rejected. The null hypothesis of the robust Hansen’s *J statistic* is that the instruments are uncorrelated with the error term and that excluded instruments are correctly excluded from the estimated equation. The excluded instruments used in the estimations for the instrumented variables are as follows (lagged values of variables (lag) refer to averages of variables for the period 2002–2007):

- Column (1). Instrumented: Urban. Excluded instruments: lag (Births attended), lag (Labour force participation rate, female)

- Column (2): Instrumented: lag (IFFT), ln GDPpc. Excluded instruments: lag (IFF/Population), lag (CPI), lag (Births attended), lag (Urban), lag (ln GDPpc)

- Column (3): Instrumented: lag (IFFT), Urban. Excluded instruments: lag (IFF/Population), lag (CPI), lag (Labour force participation rate, female), lag (Urban)

- Column (4): Instrumented: lag (IFFT), ln GDPpc. Excluded instruments: lag (IFF/Population), lag (CPI), lag (Labour force participation rate, female), lag (Births attended)

- Column (5): Instrumented: lag (IFFT). Excluded instruments: lag (IFF/Population), lag (CPI)

- Column (6): Instrumented: GIR female, ln GDPpc. Instruments: lag (Labour force participation rate, female), Births attended, lag (GDPpc)

- Column (7): Instrumented: Urban, ln GDPpc. Excluded instruments: lag (Urban), lag (ln GDPpc), Labour force participation rate, female

- Column (8): Instrumented: GIR female. Excluded instruments: ln Population, Labour force participation rate, female

Thus, taking into account the estimation results obtained from eq. (2), research findings show that, on average, an annual 1 percentage point (p.p.) increase in the ratio of IFFT would be associated with an average 0.460 p.p. decrease in the level of family planning coverage rates and an average 0.312 p.p. decrease in antenatal care coverage rates. Given that there was an estimated annual average of 837 million women aged between 15 and 49 years in the countries included in the full sample, this result suggests that around 3.9 million women would not receive this basic health care intervention as a long run effect of this increase in IFFT. Similarly, an average annual 1 p.p. increase in the ratio of IFFT would be associated with a 0.31 to 0.33 p.p. decrease in the level of infant vaccination coverage rates. It is also noteworthy that in the case of DTP3 and measles, the values of the estimated coefficients for IFFT were not far from the estimated long-run impact of IFFT on the infant vaccination coverage rate (− 0.19 p.p. in the level of coverage of DPT3, measles, and polio in combination) estimated by Ortega et al. [19], who used panel data regression methods and a sample of 56 LMICS for the period 2002 to 2013. Given that there was an estimated annual average of 65 million infants in the countries in the sample, this result suggests that around 190,000 children may not receive these basic healthcare interventions as a long-run effect of this increase in IFFT.

**Fig. 6:**
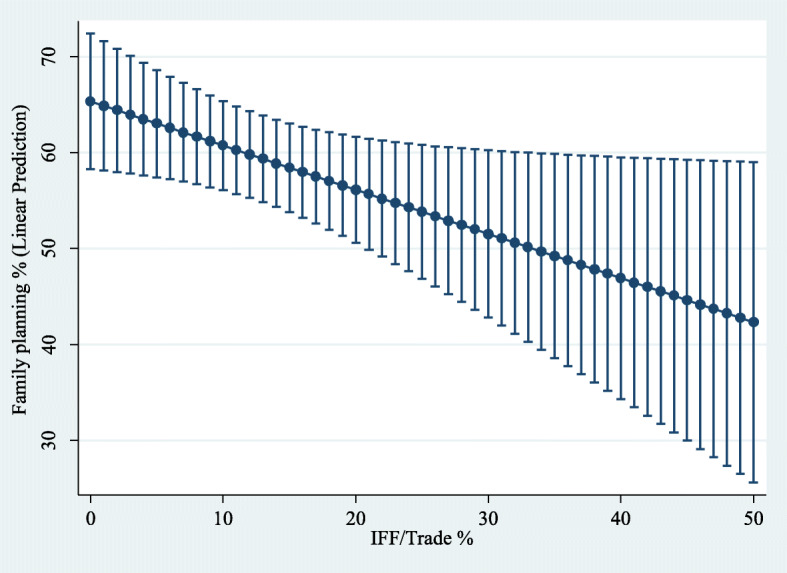
Influence of IFFT on predicted family planning coverage rates. Predictive margins (with 95% confidence intervals) while holding every independent variable constant at their sample mean

**Fig. 7:**
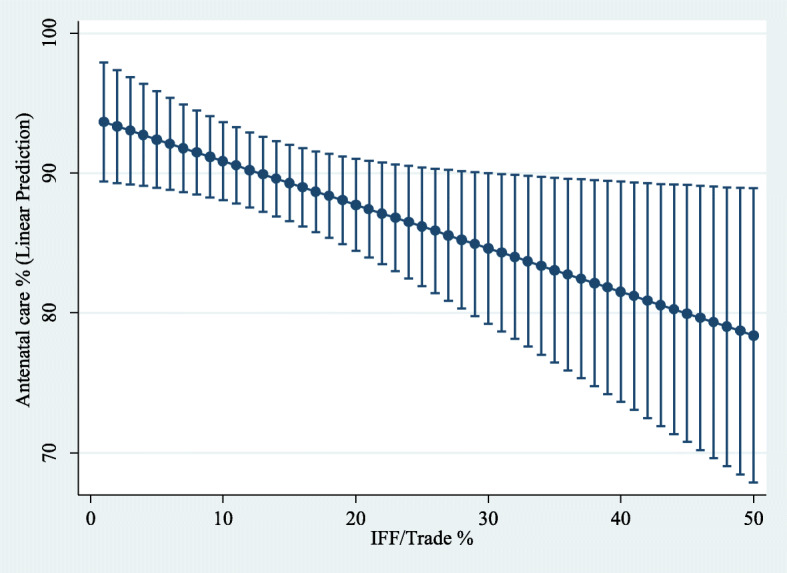
Influence of IFFT on predicted antenatal care coverage rates. Predictive margins (with 95% confidence intervals) while holding every independent variable constant at their sample mean

**Fig. 8:**
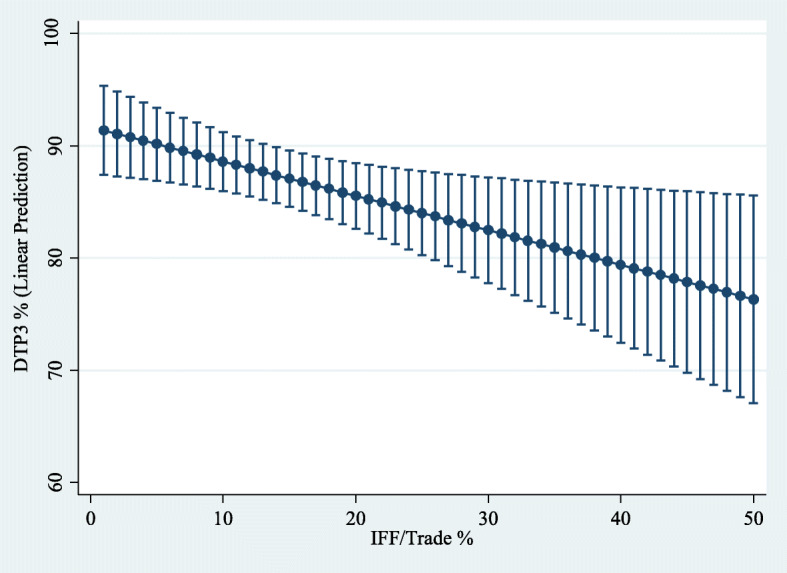
Influence of IFFT on predicted DTP3 coverage rates. Predictive margins (with 95% confidence intervals) while holding every independent variable constant at their sample mean

**Fig. 9:**
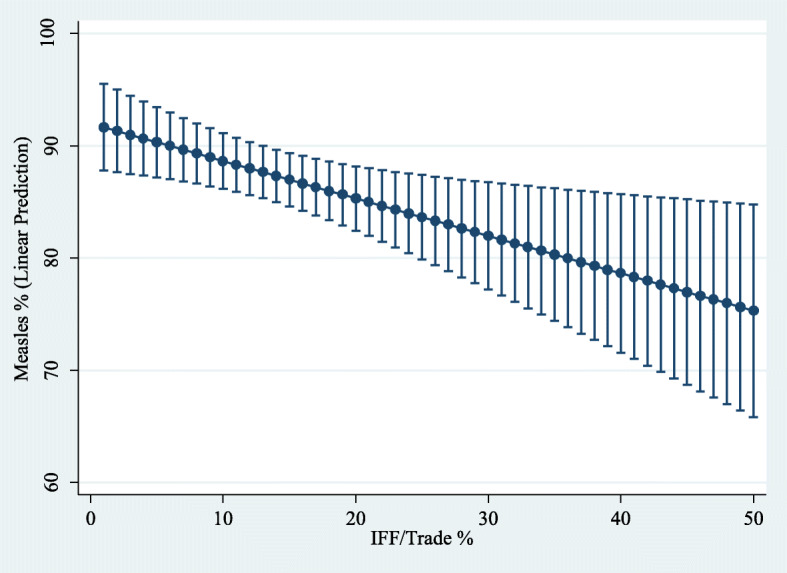
Influence of IFFT on predicted measles coverage rates. Predictive margins (with 95% confidence intervals) while holding every independent variable constant at their sample mean

The margins command in Stata was used to generate predictions of the level of dependent variables for different values of the variable of interest IFFT when the rest of the regressors in the model were evaluated at the sample mean. Next, employing the command marginsplot in Stata, a 95% confidence interval for the sample average predicted values of the dependent variables at specified values of IFFT (see Figs. 6, 7, 8 and 9). These figures show how a higher level of the variable IFFT is associated with lower estimated coverage in family planning, antenatal care, and infant immunisation coverage.

Robustness checks were also performed to assess the sensitivity of the estimation results obtained from the baseline model to the inclusion of regional effects. With this aim, a set of dummy variables was constructed to control for the region that each country belongs to (SSA, LAC, ECA, EAP MENA, and SA; see Table 2). For example, the variable SSA equals 1 for the countries which belong to the Sub-Saharan Africa region, and equals 0 otherwise. Interaction terms between these regional dummies and the variable of interest (lagIFFT) were included in the base model as regressors. Thus, the estimated model in this case is specified as follows:

3 $$ {\mathrm{ICEHS}}_{\mathrm{ji}}={\upalpha}_{\mathrm{EAP}}{\mathrm{EAP}}_{\mathrm{i}}\cdotp {\mathrm{lagIFFT}}_{\mathrm{i}}+{\upalpha}_{\mathrm{ECA}}{\mathrm{ECA}}_{\mathrm{i}}\cdotp {\mathrm{lagIFFT}}_{\mathrm{i}}+{\upalpha}_{\mathrm{LAC}}{\mathrm{LAC}}_{\mathrm{i}}\cdotp {\mathrm{lagIFFT}}_{\mathrm{i}}+{\upalpha}_{\mathrm{MENA}}{\mathrm{MENA}}_{\mathrm{i}}\cdotp {\mathrm{lagIFFT}}_{\mathrm{i}}+{\upalpha}_{\mathrm{SA}}{\mathrm{SA}}_{\mathrm{i}}\cdotp {\mathrm{lagIFFT}}_{\mathrm{i}}+{\upalpha}_{\mathrm{SSA}}{\mathrm{SSA}}_{\mathrm{i}}\cdotp {\mathrm{lagIFFT}}_{\mathrm{i}}+\upbeta\ \ln\ {\mathrm{GDPpc}}_{\mathrm{i}}+{\upvarepsilon}_{\mathrm{i}} $$

The estimation results presented in Table 6 show that there are significant regional effects and that the negative effect of the IFFs is not evenly distributed across regions. Specifically, we found that IFFs had a negative association with the variable ‘Family Planning’ only in countries belonging to ECA, MENA, and SSA regions. In the case of ‘Antenatal care’, when regional interaction terms are considered, the only region for which a significant negative effect was detected was South Asia (SA). Nevertheless, this result should be taken with caution because this coefficient was estimated with only two degrees of freedom (given that only three countries in the sample belong to this region). The estimation results also suggest that the negative association between IFFs and child immunization was limited to ECA countries. Finally, it is noteworthy that, in relation to effective tuberculosis treatment and sanitation coverage, countries in SSA region were hard hit by IFFs. Given the immense importance of these interventions for health outcomes, attention should be paid to the potential severe negative effect on health that IFFs may have in this region.

**Table 6:** 2SLS estimations. Baseline model with regional effects, eq. (3)

Independent variables	Dependent variable							
	*Family planning* (1)	*Antenatal care* (2)	*DTP3* (3)	*Measles* (4)	*Tuberculosis* (5)	*Sanitation* (6)	*ln Beds* (7)	*ln Physic* (8)
*EAP·Lag (IFFT)*	0.464 (0.691)	−0.684 (0.178)	−0.301 (0.473)	− 0.476 (0.327)	−1.146 (0.214)	− 0.882 (0.310)	− 0.047 (0.134)	−0.052 (0.449)
*ECA·Lag (IFFT)*	−1.052^c^ (0.008)	−0.181 (0.315)	−0.326^b^ (0.037)	− 0.242^b^ (0.050)	0.021 (0.949)	0.346 (0.179)	0.057^c^ (0.002)	0.086^c^ (0.007)
*LAC Lag (IFFT)*	0.451 (0.148)	−0.166 (0.303)	−0.285^a^ (0.068)	− 0.219 (0.169)	−0.074 (0.743)	− 0.032 (0.884)	−0.022^a^ (0.052)	0.033 (1.16)
*MENA Lag (IFFT)*	−0.606^c^ (0.001)	0.038 (0.670)	0.162^a^ (0.067)	0.024 (0.771)	0.300 (0.138)	−0.382^a^ (0.094)	−0.010 (0.046)	− 0.015 (0.517)
*SA Lag (IFFT)*	0.842 (0.198)	−2.409^c^ (0.000)	0.343 (0.696)	0.109 (0.866)	−1.392^a^ (0.059)	−2.365 (0.150)	−0.102^c^ (0.001)	−0.027 (0.603)
*SSA·Lag (IFF/Trade)*	−0.646^c^ (0.009)	0.133 (0.595)	0.006 (0.981)	−0.211 (0.337)	−0.547^a^ (0.058)	−2.641^c^ (0.000)	− 0.037^b^ (0.031)	−0.130^c^ (0.002)
*ln GDPpc*	7.35^c^ (0.000)	10.69^c^ (0.000)	10.33^c^ (0.000)	10.38^c^ (0.000)	6.50^c^ (0.000)	8.72^c^ (0.000)	0.08^c^ (0.000)	0.35 (0.360)
RESET test (*p*-value)	0.16 (0.688)	1.71 (0.196)	3.06 (0.085)	3.00 (0.088)	0.07 (0.792)	12.07 (0.001)	0.14 (0.701)	9.71 (0.003)
K-P rk LM (*p*-value)	21.60 (0.001)	20.23 (0.003)	21.60 (0.001)	21.60 (0.001)	23.44 (0.005)	23.44 (0.005)	23.44 (0.005)	23.25 (0.313)
K-P rk F	5.30	5.16	5.30	5.30	7.04	7.04	3.88	4.14
J statistic (*p*-value)	6.99 (0.222)	7.11 (0.212)	5.11 (0.403)	5.55 (0.353)	10.80 (0.214)	9.50 (0.302)	12.39 (0.192)	14.18 (0.165)
# Countries	72	64	72	72	71	71	62	65

Estimated coefficients and statistics p-values in brackets. ^a^ Denotes coefficient statistically significant at the 10% level, ^b^ at the 5% level, and ^c^ at the 1% level. Standard errors and covariance are consistent to various violations of conditional homoscedasticity. *RESET* is the regression equation specification error (heteroscedastic-robust) test designed to test for missing (excluded) regressors. It also has great power to detect non-linearities in the model. Thus, rejection of the null hypothesis could be due to either a nonlinearity or an omitted explanatory variable. If the heteroscedasticity-robust Kleinbergen-Paap rk LM statistic for *underidentification test* (*K-P rk LM)* rejects the null hypothesis, then the model is identified. The *weak identification test (K-P rk F)* is the robust Kleinbergen-Paap rk Wald F statistic. The null hypothesis of the robust Hansen’s *J statistic* is that the instruments are uncorrelated with the error term and that excluded instruments are correctly excluded from the estimated equation. The excluded instruments used in the estimations for the instrumented variables are as follows (lagged values of variables (lag) refer to averages of variables for the period 2002–2007):

- Columns (1), (2), (3) and (4): Instrumented: Interaction terms between the six regional dummies and lag (IFFT), ln GDPpc. Excluded instruments: Regional dummies, and interaction terms between the six regional dummies and lag (IFF/Population)

- Columns (5) and (6): Instrumented: Interaction terms between the six regional dummies and lag (IFFT), ln GDPpc. Excluded instruments: Regional dummies, and interaction terms between the six regional dummies and lag (IFF/Population), CPI, Births attended, Urban

- Column (7): Instrumented: Interaction terms between the six regional dummies and lag (IFFT), ln GDPpc. Excluded instruments: Regional dummies, and interaction terms between the six regional dummies and lag (IFF/Population), Births attended, lag (Urban), lag (ln GDPpc), Labour force participation rate, female

- Column (8): Instrumented: Interaction terms between the six regional dummies and lag (IFFT), ln GDPpc. Excluded instruments: Regional dummies, and interaction terms between the six regional dummies and lag (IFF/Population), Births attended, Dependency, lag (ln GDPpc), Density

## Discussion

Illicit financial flows are a global problem that have recently come under increased scrutiny by international policy communities, including the World Bank and the UN systems. The negative effects of increasing IFFs on development calls for the urgent need to reduce these flows, which would reduce the resource gap to finance the provision of public services and investments while simultaneously stabilizing public debt levels [61]. Given this background, this study quantified the strength of association between IFFs and the coverage of essential health-care services in LMICS.

While controlling for a range of relevant factors, the main result of the empirical analysis was that the relative level of IFFs over this period had a negative association with family planning service coverage, antenatal care service coverage, and infant DPT3 and measles vaccination coverage. These research findings also suggest that it is not simply the volume of IFFs that is curbing relevant RMNCH interventions coverage; rather, what seems to harm these ICEHS are periods in which there is an increase in the ratio of IFFs to total trade. These findings are particularly relevant given that RMNCH interventions encompass health concerns across the life course, ranging from adolescent girls and women before and during pregnancy and delivery, to newborn babies (i.e. in the first month of life), and to children.

Nevertheless, some limitations of this empirical analysis should be noted. This study uses data at the country level and it must be recognised that the negative effects of IFFs on the provision of ICEHS would likely have a greater impact on low-income rural sub-populations within countries. In this regard, Boerma et al. [62] noted that within-country inequalities in coverage have slowly fallen in most countries, and that in several countries significant poor-rich, urban-rural, or geographical gaps persist in most RMNCH indicators. Unfortunately, no data were available to analyse such a differential effect. Further research is also needed to study the effects of IFFs on the provision of other ICEHS, such as non-communicable diseases (NCD). However, this dimension of healthcare has not been addressed in this study due to lack of data. The estimation results also show that the negative influence of IFFs is not evenly distributed across regions. For this reason, the sample of countries included in the analysis must be enlarged to obtain more general results and implement specific regional analyses. Finally, it is important to note that, in a regression analysis, the estimated dependence of one variable on other variables does not necessarily imply causation: a rigorous statistical causal hypothesis test has to be grounded on an explicit and solid theoretical model. Nevertheless, social science research is frequently based on weak or incomplete theories, and its empirical generalizations are almost always the outcome of numerous iterations [63]. Taking this into account, the negative associations documented in this article could indicate that IFFs have a negative influence on the provision of basic health care interventions. However, although we consider that this effect is mainly due to reductions in the governmental fiscal resources available for investment in sectors with an impact on health outputs and outcomes (i.e. because of resource leakages in the form of IFFs), the exact channels through which this effect may occur deserve further research.

## Conclusions

The research findings suggest that IFFs are associated with damage to the provision of modern family planning methods and antenatal care. Thus, the resources lost through IFFs, and the following lack of stability of funding [64], would be associated with the fact that more than 214 million women in developing countries who want to avoid pregnancy are not using a modern contraception method [65], and that in SSA, 24% of women have an unmet need for family planning [66]. These effects are of prime importance, not only to the well-being of a population, but also to the long-term development prospects of poor countries [65, 67]. Early investment in family planning would significantly reduce unwanted pregnancies and birth rates, and yield significant savings in the costs of maternal and newborn care and immunization [68]. Furthermore, infertility is also a covert burden of reproductive health. This issue is of particular concern in LMICs, where having children is highly valued. Thus, infertility can lead to severe social stigma, economic deprivation, denial of inheritance, divorce, and social isolation. In other words, the benefits of family planning pay dividends over many years, and make it easier to achieve other development goals [69].

Furthermore, an increase in the IFFT would be associated with a decrease in the level of measles and DPT3 vaccine coverage. This is a very significant association given that immunisation tops the list of basic health care interventions of proven effectiveness in improving health status [70]. Moreover, despite immense progress during the 1980s, an estimated 19.4 million infants worldwide are still not reached by routine immunisation services. Close to 60% of these children live in Angola, the Democratic Republic of the Congo, Ethiopia, India, Indonesia, Iraq, Nigeria, Pakistan, the Philippines, and the Ukraine [71]. Thus, the loss of domestic resources through IFFs would explain why low immunisation coverage has been observed in countries experiencing high relative levels of IFFs, such as Ethiopia and the Democratic Republic of the Congo in SSA or Azerbaijan and Georgia in ECA regions, among others.

Future research should assess the specific effects of IFFs on public resources invested in health, and analyse how these flows can be curtailed such that health-related SDGs can be achieved, especially in developing countries. Further research should also analyse the observed differential regional effects and attempt to explain these findings. Nevertheless, the research findings suggest that the volume of IFFs in LMICs must be reduced in order to improve the living conditions of their populations. This objective must be addressed at the global level, not only because it is a humanitarian issue, but also because of the effects of IFFs on growth and development in LMICs. This issue becomes even more pressing if the time has come to envisage health services across countries as a global public good.

## Supplementary information

**Additional file 1.**

**Additional file 2.**

## Data Availability

All data employed are publicly available. The dataset used and analysed by the current study are available from the corresponding author on reasonable request. All documents and databases employed are available at: Global Financial Integrity: https://gfintegrity.org/ Transparency International: https://www.transparency.org/ United Nations: http://data.un.org/ and https://population.un.org/ World Bank: https://data.worldbank.org/indicator/ World Health Organization: https://www.who.int/gho/database/ World Trade Organization: https://timeseries.wto.org/

## Abbreviations

AAAA: Addis Ababa Action Agenda
CPI: Corruption Perceptions Index
DTP3: Three doses of combined diphtheria, tetanus toxoid and pertussis vaccine
EAP: East Asia & Pacific
ECA: Europe & Central Asia
FDI: Foreign direct investment
GDP: Gross Domestic Product
GFI: Global Financial Integrity
GIR: Gross intake ratio in first grade of primary education
GNI: Gross National Income
HE: Health expenditure
ICEHS: Indicators of coverage of essential health services
IFFs: Illicit financial flows
IFFT: IFFs to total trade
IV: Instrumental variables
LAC: Latin America & Caribbean
LMICs: Low- and middle-income countries
MDG: Millennium Development Goals
MENA: Middle East & North Africa
NCD: Non-communicable diseases
ODA: Official development assistance
OECD: Organisation for Economic Co-operation and Development
OLS: Ordinary least squares
RESET: The regression equation specification error test
RMNCH: Reproductive, maternal, newborn, and child health
SA: South Asia
SDGs: Sustainable Development Goals
SSA: Sub-Saharan Africa
2SLS: Two-step least squares
UHC: Universal health coverage
UN: United Nations
WHO: World Health Organization
