# Supplementary material for: Illicit financial flows and the provision of child and maternal health services in low- and middle-income countries
Source: BMC Int Health Hum Rights. 2020 Jul 11;20:15. doi: 10.1186/s12914-020-00236-w (PMC7353727; doi:10.1186/s12914-020-00236-w)
Supplement: Supplementary file 1 — Additional file 1. [file 12914_2020_236_MOESM1_ESM.docx]

| **Table A1** Number of annual observations for each country in the original database. Period 2008-2013 | | | | | | | | | | | | | | | | | |
| --- | --- | --- | --- | --- | --- | --- | --- | --- | --- | --- | --- | --- | --- | --- | --- | --- | --- |
| *Country* | *Dependent variable* | | | | | | | | *Country* | *Dependent variable* | | | | | | | |
|  | *FP* | *AC* | *M* | *DTP* | *T* | *S* | *HB* | *P* |  | *FP* | *AC* | *M* | *DTP* | *T* | *S* | *HB* | *P* |
| *Albania* | 6 | 1 | 6 | 6 | 6 | 6 | 3 | 5 | *Jordan* | 6 | 1 | 6 | 6 | 6 | 6 | 3 | 3 |
| *Algeria* | 6 | 1 | 6 | 6 | 6 | 6 | 0 | 1 | *Kazakhstan* | 6 | 1 | 6 | 6 | 6 | 6 | 3 | 6 |
| *Argentina* | 6 | 1 | 6 | 6 | 6 | 6 | 3 | 2 | *Lao PDR* | 6 | 2 | 6 | 6 | 6 | 6 | 2 | 3 |
| *Armenia* | 6 | 1 | 6 | 6 | 6 | 6 | 3 | 5 | *Lesotho* | 6 | 1 | 6 | 6 | 6 | 6 | 0 | 0 |
| *Azerbaijan* | 6 | 1 | 6 | 6 | 6 | 6 | 3 | 5 | *Madagascar* | 6 | 2 | 6 | 6 | 6 | 6 | 1 | 5 |
| *Bangladesh* | 6 | 3 | 6 | 6 | 6 | 6 | 1 | 5 | *Malawi* | 6 | 1 | 6 | 6 | 6 | 6 | 1 | 3 |
| *Belarus* | 6 | 1 | 6 | 6 | 6 | 6 | 2 | 6 | *Malaysia* | 6 | 4 | 6 | 6 | 6 | 6 | 4 | 3 |
| *Belize* | 6 | 1 | 6 | 6 | 6 | 6 | 5 | 2 | *Maldives* | 6 | 1 | 6 | 6 | 6 | 6 | 1 | 2 |
| *Botswana* | 6 | 0 | 6 | 6 | 6 | 6 | 2 | 4 | *Mali* | 6 | 2 | 6 | 6 | 6 | 6 | 2 | 3 |
| *Brazil* | 6 | 1 | 6 | 6 | 6 | 6 | 4 | 5 | *Mexico* | 6 | 2 | 6 | 6 | 6 | 6 | 4 | 6 |
| *Bulgaria* | 6 | 0 | 6 | 6 | 6 | 6 | 4 | 5 | *Moldova* | 6 | 1 | 6 | 6 | 6 | 6 | 3 | 4 |
| *Burkina Faso* | 6 | 1 | 6 | 6 | 6 | 6 | 1 | 5 | *Morocco* | 6 | 1 | 6 | 6 | 6 | 6 | 2 | 2 |
| *Cabo Verde* | 6 | 0 | 6 | 6 | 6 | 6 | 2 | 4 | *Namibia* | 6 | 1 | 6 | 6 | 6 | 6 | 1 | 1 |
| *Cambodia* | 6 | 1 | 6 | 6 | 6 | 6 | 2 | 6 | *Nicaragua* | 6 | 1 | 6 | 6 | 6 | 6 | 4 | 6 |
| *Cameroon* | 6 | 1 | 6 | 6 | 6 | 6 | 1 | 2 | *Niger* | 6 | 1 | 6 | 6 | 6 | 6 | 0 | 2 |
| *Chad* | 6 | 1 | 6 | 6 | 6 | 6 | 0 | 2 | *Panama* | 6 | 2 | 6 | 6 | 6 | 6 | 3 | 6 |
| *China* | 6 | 6 | 6 | 6 | 6 | 6 | 3 | 4 | *Paraguay* | 6 | 1 | 6 | 6 | 6 | 6 | 4 | 1 |
| *Colombia* | 6 | 1 | 6 | 6 | 6 | 6 | 2 | 3 | *Peru* | 6 | 4 | 6 | 6 | 6 | 6 | 5 | 3 |
| *Congo, Rep.* | 6 | 1 | 6 | 6 | 6 | 6 | 1 | 1 | *Philippines* | 6 | 3 | 6 | 6 | 6 | 6 | 3 | 0 |
| *Costa Rica* | 6 | 2 | 6 | 6 | 6 | 6 | 4 | 1 | *Romania* | 6 | 0 | 6 | 6 | 6 | 6 | 3 | 4 |
| *Cote d'Ivoire* | 6 | 1 | 6 | 6 | 6 | 6 | 0 | 2 | *Russian F.* | 6 | 0 | 6 | 6 | 6 | 6 | 0 | 4 |
| *Croatia* | 6 | 5 | 6 | 6 | 6 | 6 | 5 | 5 | *S. Tome P.* | 6 | 1 | 6 | 6 | 6 | 6 | 1 | 0 |
| *Djibouti* | 6 | 1 | 6 | 6 | 6 | 6 | 2 | 1 | *Serbia* | 6 | 1 | 6 | 6 | 6 | 6 | 1 | 5 |
| *Dominican R* | 6 | 1 | 6 | 6 | 6 | 6 | 4 | 2 | *Sierra L.* | 6 | 3 | 6 | 6 | 6 | 6 | 0 | 2 |
| *Ecuador* | 6 | 0 | 6 | 6 | 6 | 6 | 4 | 3 | *South A.* | 6 | 1 | 6 | 6 | 6 | 6 | 0 | 4 |
| *El Salvador* | 6 | 1 | 6 | 6 | 6 | 6 | 5 | 2 | *Sudan* | 6 | 1 | 6 | 6 | 6 | 6 | 3 | 1 |
| *Ethiopia* | 6 | 1 | 6 | 6 | 6 | 6 | 2 | 3 | *Suriname* | 6 | 1 | 6 | 6 | 6 | 6 | 2 | 0 |
| *Gambia, The* | 6 | 2 | 6 | 6 | 6 | 6 | 2 | 2 | *Swaziland* | 6 | 1 | 6 | 6 | 6 | 6 | 1 | 1 |
| *Georgia* | 6 | 1 | 6 | 6 | 6 | 6 | 3 | 6 | *Tanzania* | 6 | 2 | 6 | 6 | 6 | 6 | 1 | 2 |
| *Guatemala* | 6 | 1 | 6 | 6 | 6 | 6 | 4 | 1 | *Thailand* | 6 | 2 | 6 | 6 | 6 | 6 | 1 | 3 |
| *Guinea.Bissau* | 6 | 1 | 6 | 6 | 6 | 6 | 1 | 3 | *Togo* | 6 | 1 | 6 | 6 | 6 | 6 | 1 | 2 |
| *Guyana* | 6 | 1 | 6 | 6 | 6 | 6 | 1 | 2 | *Tunisia* | 6 | 1 | 6 | 6 | 6 | 6 | 4 | 3 |
| *Haiti* | 6 | 1 | 6 | 6 | 6 | 6 | 0 | 0 | *Ukraine* | 6 | 1 | 6 | 6 | 6 | 6 | 2 | 4 |
| *Honduras* | 6 | 1 | 6 | 6 | 6 | 6 | 5 | 0 | *Venezuela* | 6 | 0 | 6 | 6 | 6 | 6 | 2 | 0 |
| *India* | 6 | 1 | 6 | 6 | 6 | 6 | 1 | 5 | *Vietnam* | 6 | 1 | 6 | 6 | 6 | 6 | 3 | 6 |
| *Indonesia* | 6 | 5 | 6 | 6 | 6 | 6 | 2 | 3 | *Zambia* | 6 | 0 | 6 | 6 | 6 | 6 | 2 | 5 |
| *Jamaica* | 6 | 2 | 6 | 6 | 6 | 6 | 3 | 1 | ***Number*** | 73 | 65 | 73 | 73 | 73 | 73 | 64 | 66 |
| Notes: Family Planning (FP); Antenatal care (AC); Measles (M); DPT3 (DTP); Tuberculosis (T); Sanitation (S); Hospital Beds (HB); Physicians (P). ***Number*** refers to the number of countries with at least one annual observation in the period (i.e. # of cross–section employed in the regression analysis for each variable). | | | | | | | | | | | | | | | | | |

**Table A2** Correlation matrix of dependent and independent variables

|  | 1. | 2. | 3. | 4. | 5. | 6. | 7. | 8. | 9. | 10. | 11. |
| --- | --- | --- | --- | --- | --- | --- | --- | --- | --- | --- | --- |
| 1. *Family planning* | 1.00 |  |  |  |  |  |  |  |  |  |  |
| 2. *Antenatal care* | 0.21 | 1.00 |  |  |  |  |  |  |  |  |  |
| 3. *DTP3* | 0.20 | 0.51 | 1.00 |  |  |  |  |  |  |  |  |
| 4. *Measles* | 0.28 | 0.56 | 0.89 | 1.00 |  |  |  |  |  |  |  |
| 5. *Tuberculosis* | 0.22 | 0.32 | 0.47 | 0.52 | 1.00 |  |  |  |  |  |  |
| 6. *Sanitation* | 0.36 | 0.66 | 0.57 | 0.67 | 0.47 | 1.00 |  |  |  |  |  |
| 7. *ln Hospitals beds* | 0.11 | 0.40 | 0.23 | 0.37 | 0.38 | 0.55 | 1.00 |  |  |  |  |
| 8. *ln Physicians* | 0.39 | 0.53 | 0.41 | 0.56 | 0.56 | 0.75 | 0.61 | 1.00 |  |  |  |
| 9. *lag (IFF/Trade)* | -0.15 | -0.13 | -0.15 | -0.24 | 0.08 | -0.12 | -0.00 | -0.09 | 1.00 |  |  |
| 10*. ln GDPpc* | 0.43 | 0.57 | 0.42 | 0.56 | 0.45 | 0.72 | 0.59 | 0.83 | -0.18 | 1.00 |  |
| 11. *GIR, female* | 0.21 | -0.17 | -0.18 | -0.31 | -0.14 | -0.33 | -0.24 | -0.21 | 0.18 | -0.35 | 1.00 |
| 12. *Urban* | 0.25 | 0.50 | 0.26 | 0.31 | 0.38 | 0.60 | 0.35 | 0.63 | 0.02 | 0.63 | -0.28 |

Notes: coefficient of correlations computed employing a common sample of 50 observations for the period 2008-2013. Lagged values of variables refer to values of variables for the previous period 2002-2007.
