# Supplementary material for: Illicit financial flows and the provision of child and maternal health services in low- and middle-income countries
Source: BMC Int Health Hum Rights. 2020 Jul 11;20:15. doi: 10.1186/s12914-020-00236-w (PMC7353727; doi:10.1186/s12914-020-00236-w)
Supplement: Supplementary file 2 — Additional file 2. [file 12914_2020_236_MOESM2_ESM.docx]

**Testing the exogeneity and relevance of the excluded instruments employed in the estimations**

As discussed in the main text, an instrumental variable must not be correlated with the regression’s error (it must be exogenous) and it must be highly correlated with the included endogenous regressors (it must be relevant). In order to explain how the employed instruments sets meets IV assumptions, we provide additional details on the test statistics and procedures used in the article to assess the reliability of the 2SLS regressions. Thus, as an example of the procedures employed, we may consider the estimations results presented in Table 4, column (1). In this estimation, the process of selection and validation of the instruments set can be described as follows:

1. We studied which of the two independent variables in the model - *lag (IFF/Trade)* and *ln GDPpc* - could be considered exogenous. This was done by means of applying an endogeneity test (endog()option in ivreg2 routine in Stata). In this case, the null hypothesis of exogeneity was rejected only in the case of the variable *ln GDPpc*.
2. Then we selected the instruments employed in the 2SLS estimation. Considering that the exogenous regressor (*lag IFF/Trade*) is serving as its own instrument, the instrument set in this case included *lag (Urban)* and *lag (ln GDPpc)* as excluded instruments. We chose these variables as potential valid excluded instruments because prior knowledge suggests that these variables are potentially exogenous, and the correlation coefficients between these variables and the endogenous regressor *ln GDPpc* are high (as was the case, 0.70 and 0.99 respectively, see Table A3).
3. The next step is to study the validity (relevance and exogeneity) of the excluded instruments set. On the one hand, the reported value of the *J statistic* (1.11) indicated that we cannot to reject the null hypothesis that the instruments are uncorrelated with the error term, and that they are correctly excluded from the estimated equation (i.e. the instruments set was exogenous). On the other hand, the reported value heteroscedasticity-robust *Kleinbergen-Paap rk LM statistic* (46.7) rejected the null hypothesis that the model was underidentified (i.e. the instruments were relevant in the sense that there was sufficient correlation between the instruments and the endogenous regressor to guarantee that the 2SLS estimator produced unique estimates). Besides this, the reported value of the robust *Kleinbergen-Paap rk Wald F statistic* (88,672) suggested that the instruments set was not weak (i.e. the instruments were sufficiently correlated with the endogenous regressor). This last test is very important given that if the instruments used are weak, the 2SLS estimator is biased and the t statistics and the estimated confidence intervals are not reliable.

Please insert Table A3 about here

This process of both detecting endogenous regressors and of selecting and validating the instruments set employed in the estimations was applied in all regressions included in Tables 4 and 5. In the case of Table 6 (baseline model with regional effects), we considered all the independent variables to be endogenous. In addition to this, we may also test the relevance of instruments by examining the fit of the first-stage regressions of the endogenous regressors on the full set of exogenous excluded instruments. One rule of thumb is that the *F-statistic* from the first stage regression for each endogenous should be at least 10 for weak identification not to be considered a problem. Also, if an estimated first-stage equation produces a large value of the *standard partial R^2^* and a small value of the *Shea partial R^2^*, the instruments employed lack sufficient relevance to explain the endogenous (instrumented) regressor. The following Tables A4 to A6 show the summary results for the first-stage regressions corresponding to Tables 5 to 6 in the main text.

Please insert Tables A4, A5 and A6 about here

In the light of the results presented in Tables A4 to A6, no weak-instrument problem was detected in the regressions (with perhaps the exception of equation (7) in Table 6).

**Table A3** Correlation matrix of independent variables and excluded instruments

|  | 1. | 2. | 3. | 4. | 5. | 6. | 7. | 8. | 9. | 10. | 11. | 12. | 13. | 14. | 15. | 16. | 17. | 18. |
| --- | --- | --- | --- | --- | --- | --- | --- | --- | --- | --- | --- | --- | --- | --- | --- | --- | --- | --- |
| 1. *lag (IFF /Trade)* | 1.00 |  |  |  |  |  |  |  |  |  |  |  |  |  |  |  |  |  |
| 2*. ln GDPpc* | -0.19 | 1.00 |  |  |  |  |  |  |  |  |  |  |  |  |  |  |  |  |
| 3. *GIR, female* | 0.21 | -0.28 | 1.00 |  |  |  |  |  |  |  |  |  |  |  |  |  |  |  |
| 4. *Urban* | 0.00 | 0.70 | -0.23 | 1.00 |  |  |  |  |  |  |  |  |  |  |  |  |  |  |
| 5. *Births attended* | -0.12 | 0.82 | -0.29 | 0.66 | 1.00 |  |  |  |  |  |  |  |  |  |  |  |  |  |
| 6. *CPI* | -0.09 | 0.35 | -0.06 | 0.25 | 0.37 | 1.00 |  |  |  |  |  |  |  |  |  |  |  |  |
| 7. *Density* | -0.13 | -0.04 | 0.04 | -0.18 | -0.12 | -0.13 | 1.00 |  |  |  |  |  |  |  |  |  |  |  |
| 8. *Depen-dency* | 0.11 | -0.78 | 0.09 | -0.51 | -0.74 | -0.23 | -0.18 | 1.00 |  |  |  |  |  |  |  |  |  |  |
| 9. *IFF/ GDP* | 0.74 | -0.12 | 0.06 | 0.02 | 0.00 | 0.08 | -0.10 | 0.02 | 1.00 |  |  |  |  |  |  |  |  |  |
| 10. *LFPR, female* | 0.15 | -0.46 | 0.44 | -0.44 | -0.38 | -0.21 | 0.02 | 0.36 | 0.07 | 1.00 |  |  |  |  |  |  |  |  |
| 11. *lag (Births attend.)* | -0.08 | 0.82 | -0.25 | 0.70 | 0.94 | 0.40 | -0.12 | -0.74 | 0.05 | -0.38 | 1.00 |  |  |  |  |  |  |  |
| 12. *lag (CPI)* | -0.21 | 0.46 | -0.15 | 0.38 | 0.44 | 0.87 | -0.12 | -0.29 | 0.06 | -0.26 | 0.48 | 1.00 |  |  |  |  |  |  |
| 13. *lag (ln GDPpc)* | -0.19 | 0.99 | -0.28 | 0.72 | 0.81 | 0.36 | -0.06 | -0.73 | -0.11 | -0.48 | 0.82 | 0.47 | 1.00 |  |  |  |  |  |
| 14. *lag (IFF/ GDP)* | 0.86 | -0.01 | -0.04 | 0.11 | 0.12 | 0.04 | -0.14 | -0.01 | 0.84 | -0.01 | 0.14 | 0.01 | 0.01 | 1.00 |  |  |  |  |
| 15. *lag (IFF/ Popul.)* | 0.44 | 0.57 | -0.18 | 0.38 | 0.48 | 0.31 | -0.13 | -0.37 | 0.43 | -0.19 | 0.49 | 0.33 | 0.58 | 0.60 | 1.00 |  |  |  |
| 16. *lag (Urban)* | 0.02 | 0.70 | -0.23 | 0.99 | 0.66 | 0.24 | -0.22 | -0.51 | 0.02 | -0.45 | 0.71 | 0.36 | 0.72 | 0.12 | 0.39 | 1.00 |  |  |
| 17. *lag (LFPR, female)* | 0.15 | -0.45 | 0.44 | -0.45 | -0.37 | -0.21 | 0.00 | 0.31 | 0.06 | 0.98 | -0.37 | -0.28 | -0.47 | -0.03 | -0.20 | -0.47 | 1.00 |  |
| 18. *ln Popula-tion* | -0.25 | -0.03 | 0.16 | -0.04 | -0.20 | -0.16 | 0.09 | -0.08 | -0.34 | 0.03 | -0.19 | -0.21 | -0.06 | -0.43 | -0.31 | -0.06 | 0.08 | 1.00 |
| 19. *lag GDPpc* | -0.16 | 0.91 | -0.23 | 0.64 | 0.69 | 0.38 | -0.10 | -0.61 | -0.08 | -0.38 | 0.73 | 0.47 | 0.92 | -0.00 | 0.64 | 0.65 | -0.38 | -0.03 |

Note: coefficient of correlations computed employing a common sample of 66 observations for the period 2008-2013. Lagged values of variables refer to values of variables for the previous period 2002-2007.

**Table A4** Summary results (heteroscedasticity-robust) for 2SLS first-stage regressions. Baseline model (Table 4 in main text).

| Dependent  variables | Instrumented (endogenous)  variables | Shea  Partial R^2^ | Partial R^2^ | F-statistic | # Countries |
| --- | --- | --- | --- | --- | --- |
| *Family planning* (1) | *ln GDPpc* | 0.99 | 0.99 | 88·10^3^ | 72 |
| *Antenatal care* (2) | *Lag (IFF/Trade)* | 0.23 | 0.67 | 39.3 | 64 |
|  | *ln GDPpc* | 0.35 | 0.99 | 97·10^3^ |  |
| *DTP3* (3)  *Measles* (4) | *Lag (IFF/Trade)* | 0.21 | 0.21 | 16.5 | 72 |
| *Tuberculosis* (5) | *ln GDPpc* | 0.99 | 0.99 | 79 ·10^3^ | 59 |
| *Sanitation* (6) | *Lag (IFF/Trade)* | 0.76 | 0.76 | 81.8 | 72 |
| *ln Beds* (7) | *Lag (IFF/Trade)* | 0.78 | 0.91 | 132.0 | 63 |
|  | *ln GDPpc* | 0.86 | 0.99 | 1.1 · 10^5^ |  |
| *ln Physic* (8) | *Lag (IFF/Trade)* | 0.76 | 0.76 | 87.9 | 66 |

**Table A5** Summary results (heteroscedasticity-robust) for 2SLS first-stage regressions. Baseline model with additional controls (Table 5 in main text).

| Dependent  variables | Instrumented (endogenous)  variables | Shea  Partial R^2^ | Partial R^2^ | F-statistic | # Countries |
| --- | --- | --- | --- | --- | --- |
| *Family planning* (1) | *Urban* | 0.27 | 0.27 | 24.1 | 67 |
| *Antenatal care* (2) | *Lag (IFF/Trade)* | 0.40 | 0.41 | 10.2 | 60 |
|  | *ln GDPpc* | 0.97 | 0.99 | 1.4·10^3^ |  |
| *DTP3* (3) | *Lag (IFF/Trade)* | 0.40 | 0.41 | 12.2 | 67 |
|  | *Urban* | 0.95 | 0.98 | 800.7 |  |
| *Measles* (4) | *Lag (IFF/Trade)* | 0.41 | 0.41 | 13.0 | 67 |
|  | *ln GDPpc* | 0.43 | 0.43 | 10.0 |  |
| *Tuberculosis* (5) | *Lag (IFF/Trade)* | 0.37 | 0.37 | 26.3 | 67 |
| *Sanitation* (6) | *GIR female* | 0.33 | 0.71 | 36.0 | 66 |
|  | *ln GDPpc* | 0.37 | 0.78 | 92.2 |  |
| *ln Beds* (7) | *Urban* | 0.97 | 0.99 | 2.5 · 10^3^ | 60 |
|  | *ln GDPpc* | 0.98 | 0.99 | 5.0 · 10^3^ |  |
| *ln Physic* (8) | *GIR female* | 0.36 | 0.36 | 13.0 | 62 |

**Table A6** Summary results (heteroscedasticity-robust) for 2SLS first-stage regressions. Baseline model with regional effects (Table 6 in main text).

| Dependent  variables | Instrumented (endogenous)  variables | Shea  Partial R^2^ | Partial R^2^ | F-statistic | # Countries |
| --- | --- | --- | --- | --- | --- |
| *Family planning* (1),  *DTP3* (3),  *Measles* (4) | *EAP · Lag (IFF/Trade)* | 0.70 | 0.91 | 632.6 | 72 |
|  | *ECA · Lag (IFF/Trade)* | 0.58 | 0.84 | 23.8 |  |
|  | *LAC · Lag (IFF/Trade)* | 0.52 | 0.81 | 27.9 |  |
|  | *MENA · Lag (IFF/Trade)* | 0.87 | 0.94 | 48.7 |  |
|  | *SA · Lag (IFF/Trade)* | 0.83 | 0.91 | 4.4 · 10^3^ |  |
|  | *SSA · Lag (IFF/Trade)* | 0.38 | 0.65 | 17.3 |  |
|  | *ln GDPpc* | 0.47 | 0.99 | 8.7 · 10^5^ |  |
| *Antenatal care* (2) | *EAP · Lag (IFF/Trade)* | 0.69 | 0.91 | 616.8 | 64 |
|  | *ECA · Lag (IFF/Trade)* | 0.60 | 0.84 | 17.2 |  |
|  | *LAC · Lag (IFF/Trade)* | 0.52 | 0.80 | 23.5 |  |
|  | *MENA · Lag (IFF/Trade)* | 0.87 | 0.94 | 47.5 |  |
|  | *SA · Lag (IFF/Trade)* | 0.82 | 0.91 | 4.3 · 10^3^ |  |
|  | *SSA · Lag (IFF/Trade)* | 0.41 | 0.67 | 15.6 |  |
|  | *ln GDPpc* | 0.49 | 0.99 | 8.5·10^5^ |  |
| *Tuberculosis* (5),  *Sanitation* (6) | *EAP · Lag (IFF/Trade)* | 0.80 | 0.92 | 191.6 | 71 |
|  | *ECA · Lag (IFF/Trade)* | 0.65 | 0.84 | 7.6 |  |
|  | *LAC · Lag (IFF/Trade)* | 0.61 | 0.81 | 4.8 |  |
|  | *MENA · Lag (IFF/Trade)* | 0.91 | 0.94 | 10.6 |  |
|  | *SA · Lag (IFF/Trade)* | 0.87 | 0.91 | 820.0 |  |
|  | *SSA · Lag (IFF/Trade)* | 0.51 | 0.70 | 3.7 |  |
|  | *ln GDPpc* | 0.61 | 0.99 | 4.6 · 10^6^ |  |
| *ln Beds* (7) | *EAP · Lag (IFF/Trade)* | 0.88 | 0.93 | 285.1 | 62 |
|  | *ECA · Lag (IFF/Trade)* | 0.81 | 0.87 | 4.7 |  |
|  | *LAC · Lag (IFF/Trade)* | 0.75 | 0.83 | 5.8 |  |
|  | *MENA · Lag (IFF/Trade)* | 0.96 | 0.96 | 34.5 |  |
|  | *SA · Lag (IFF/Trade)* | 0.91 | 0.92 | 832.0 |  |
|  | *SSA · Lag (IFF/Trade)* | 0.68 | 0.73 | 4.0 |  |
|  | *ln GDPpc* | 0.82 | 0.99 | 2.7 · 10^7^ |  |
| *ln Physic* (8) | *EAP · Lag (IFF/Trade)* | 0.87 | 0.91 | 159.1 | 65 |
|  | *ECA · Lag (IFF/Trade)* | 0.83 | 0.87 | 8.0 |  |
|  | *LAC · Lag (IFF/Trade)* | 0.73 | 0.77 | 4.0 |  |
|  | *MENA · Lag (IFF/Trade)* | 0.94 | 0.94 | 9.8 |  |
|  | *SA · Lag (IFF/Trade)* | 0.95 | 0.96 | 4.2 · 10^3^ |  |
|  | *SSA · Lag (IFF/Trade)* | 0.85 | 0.85 | 14.5 |  |
|  | *ln GDPpc* | 0.87 | 0.99 | 5.3 · 10^7^ |  |
